# A systematic literature review (SLR) on the adoption of artificial intelligence-assisted SLRS: implications for health technology assessments

**DOI:** 10.1017/S0266462326103535

**Published:** 2026-02-16

**Authors:** Seye Abogunrin, Yifei Liu, Clarissa Higuchi Zerbini

**Affiliations:** 1 F Hoffmann-La Roche Ltd, Switzerland; 2 Department of Health Policy, London School of Economics and Political Science, UK

**Keywords:** artificial intelligence, machine learning, systematic review, health technology assessment, healthcare decision making

## Abstract

**Objectives:**

Systematic literature reviews (SLRs) are essential for evidence synthesis in healthcare decision making, including health technology assessment (HTA), but their time and resource demands are substantial. Artificial intelligence (AI) may enhance efficiency of conducting SLRs, but its acceptance by HTA bodies remains underexplored. This SLR quantifies published health-related SLRs reporting AI use, identifies AI tools used at each SLR stage, and evaluates HTA guidance on AI in evidence synthesis.

**Methods:**

We searched Embase, Medline, and the Cochrane Library (up to 9 September 2025), supplemented by hand searches and reviews of HTA agency websites. Titles and abstracts were screened in Rayyan by a single reviewer, with full-text review confirming eligibility. Data were extracted and synthesized narratively along key themes.

**Results:**

In total, 112 studies covering 111 unique SLRs were identified, reporting 134 implementations of 45 unique AI tools (29 publicly available; 16 custom-built). AI use has risen since 2013 and was most frequently applied during title and abstract screening (88 of the 134 implementations). Human oversight remained essential, with no fully autonomous AI reported. Three HTA agencies (CDA-AMC, IQWiG, NICE), EUnetHTA, JBI and Cochrane have provided guidance, indicating the formal integration of AI into HTA processes.

**Conclusions:**

This SLR provides a quantitative overview of AI use in health-related SLRs and current HTA guidance. These findings may inform development of clearer methodological recommendations and support integration of AI-assisted evidence synthesis in HTA submissions. Further research and policy development are needed to optimize its role in evidence synthesis and healthcare decision making.

## Introduction

Health technology assessment (HTA) is a multidisciplinary process that evaluates the safety, effectiveness, and cost-effectiveness of health technologies (1). By considering clinical, economic, social, ethical, and organizational aspects, HTAs inform decisions on adopting, reimbursing, or implementing new and existing technologies (1). Systematic literature reviews (SLRs) are the gold standard for evidence synthesis in HTA and inform dossiers prepared by health technology developers for review by HTA bodies as part of their decision-making process (2). Their transparent, reproducible methodology ensures comprehensive evaluation of all relevant literature for a given indication (3), and they serve as a precursor to meta-analyses and indirect comparisons (4).

SLRs involve several complex stages, including defining research questions and search strategies, developing a protocol, screening studies, extracting data, assessing risk of bias, and synthesizing findings (3). They require substantial time, budget, and human resources, and typically take over 67 weeks to complete (5). The lengthy process can render findings outdated by the time of publication (6) and delay decision-making and patient access to treatments. HTA bodies require that SLR searches are performed within 3–6 months of dossier submission, adding further logistical challenges for researchers (2;7). SLRs also demand significant financial resources, requiring expert teams, access to specialized databases and software, and periodic updates to maintain relevance (5;6;8). Consequently, a single SLR can cost up to US$141,194.80 (9). Despite these challenges, methodological rigor remains critical for minimizing bias and supporting evidence-based HTA decisions (3).

Artificial intelligence (AI), the simulation of human intelligence for tasks that involve learning, reasoning, and prediction (10), has the potential to alleviate the burden of repetitive tasks while preserving human oversight (11). Researchers have explored the use of AI to streamline and expedite various steps of the SLR process (12). Advances in natural language processing (NLP) and machine learning (ML) have enhanced the feasibility of integrating AI into SLRs and evidence synthesis (13). NLP, including large language models (LLMs) such as ChatGPT, enables machines to interpret and generate human language, and ML allows systems to learn from data (14). These technologies could enable faster, more efficient evidence synthesis to support timely decision making (13).

Understanding how HTA bodies view AI-assisted evidence synthesis for HTA submissions is crucial for developing recommendations for its use. Although some agencies have issued recommendations (15–18), many have not defined their position on the adoption of AI technologies in the evidence-synthesis process. Slow adoption of AI by HTA bodies could hinder the exploration and implementation of more dynamic HTA approaches, such as living HTAs and living reviews, which could also be assisted by AI. Furthermore, the increasing workload due to the new EU HTA Regulation (Regulation [EU] 2021/2282), which introduces joint clinical assessments across EU member states and requires consideration of multiple national PICOs within a single assessment, could be better managed using AI (7;19;20).

This study aimed to (i) quantify published health-related SLRs reporting AI use; (ii) identify AI tools used at each SLR stage and their functionalities; and (iii) determine if HTA agencies accept or recommend the use of AI tools. By addressing these questions, the study seeks to clarify the extent of AI adoption in evidence synthesis and its implications for HTA decision making; it does not examine specific AI methodologies used in SLRs (e.g., ML, deep learning, neural networks) because these have been covered in previous research (21).

## Methods

To identify AI-supported SLRs within the field of health-related research, an SLR was conducted following the Preferred Reporting Items for Systematic Reviews and Meta-Analyses (PRISMA) 2020 guidelines (22). This included structured searches across multiple databases, narrative synthesis, and risk-of-bias assessment using the Risk Of Bias In Systematic Reviews (ROBIS) tool. The protocol for this SLR was preregistered with the Open Science Framework (protocol identifier: osf.io/6tavf).

### Information sources and search strategy

Three databases were searched to identify relevant AI-supported SLRs: Embase (via Ovid), Medline (via Ovid), and the Cochrane Library (Cochrane Reviews), covering all records from database inception to 25 June 2024, with updated searches conducted on 9 September 2025. To ensure comprehensive coverage, the bibliography lists of included studies were manually reviewed between 17 June and 5 July 2024, and between 3 October and 7 October 2025, to identify potentially eligible articles that may have been missed by the database searches.

The search strategy incorporated key terms related to AI and systematic reviews. The primary keyword combination included (“artificial intelligence” OR “machine learning” OR “deep learning” OR “automation” OR “text mining” OR “natural language processing” OR “large language model” OR “generative AI”) AND “systematic review.” Additionally, the “artificial intelligence” query string was combined with the names of AI tools specifically developed to assist with SLRs, identified through prior knowledge and online searches (Supplementary Tables 1 and 2).

Only articles involving human participants and review articles were included, with the search limited to English-language publications.

#### Supplementary searches

To identify guidelines from HTA agencies on the use of AI tools for SLRs, the methodological guidelines or guidance sections of HTA agency websites were systematically searched between 17 June and 5 July 2024, and between 3 October and 8 October 2025. This included all fifty-three members of the International Network of Agencies for Health Technology Assessment (INAHTA), with the full member list available at https://www.inahta.org/members/members_list/. The website of the European Network for Health Technology Assessment (EUnetHTA), which is not a member of INAHTA, was reviewed to capture any relevant guidance.

Broader methodological frameworks commonly referenced by HTA bodies were also considered. These included the Cochrane Handbook (23), Centre for Reviews and Dissemination guidance (24), and the Joanna Briggs Institute Manual for Evidence Synthesis (25).

### Eligibility criteria

Owing to the nature of the research questions, the eligibility criteria based on the PICO framework (Population, Intervention, Comparator, Outcomes) were not suitable for this analysis. Instead, a set of concept-driven eligibility criteria relevant to the study objectives were applied to ensure a comprehensive yet focused selection of relevant studies.

Eligible articles included SLRs or reviews that employed a systematic approach, focused on human health and medical research, and reported the use of AI tools to assist in the literature review process. Studies were excluded if they only mentioned the use of reference management or screening platforms, such as Covidence or Rayyan, without confirming the use of their AI or ML functionalities. Conversely, studies using software inherently designed for AI-based active learning (e.g., ASReview) were included because its core screening functionality relies on ML algorithms. Conference abstracts, review protocols, and preprint articles were excluded to maintain a focus on fully peer-reviewed published studies.

### Overview of the screening process

Articles from database searches were first deduplicated in EndNote, then further deduplicated in Rayyan. Screening was conducted in Rayyan, beginning with title and abstract screening based on the prespecified eligibility criteria. Full texts of the studies that met the inclusion criteria were reviewed to confirm eligibility before data extraction. Screening, full-text review, and data extraction were conducted collaboratively by multiple reviewers.

### Data extraction

The following data were extracted in an Excel sheet using Rayyan: authors, article title, year of publication, type of author affiliations, type of review, topic of review, name of the AI tools used, functionalities of the AI tools, stages of review for which the AI tool was employed, whether human reviewers were involved, and the advantages and disadvantages of using the tool.

Tools designed for automating meta-analyses were beyond the scope of this review and, therefore, were not included. In cases when the functionalities and features of an AI tool were unclear, the tool’s official website was consulted for clarification. A narrative (descriptive) synthesis was conducted along key themes identified from extracted information such as reported AI tools, functionalities, and stages of use.

### Risk-of-bias assessment

The ROBIS tool was used to assess the risk of bias in the included reviews (Supplement A). This tool assesses bias across four domains: study eligibility criteria, identification and selection of studies, data collection and study appraisal, and synthesis and findings. Additionally, ROBIS provides an overall assessment of the risk of bias and limitations based on these domains (26). A high risk of bias indicates lower quality, which could be due to how the review was designed or reported.

## Results

### Overview of SLR results

**Figure 1.**
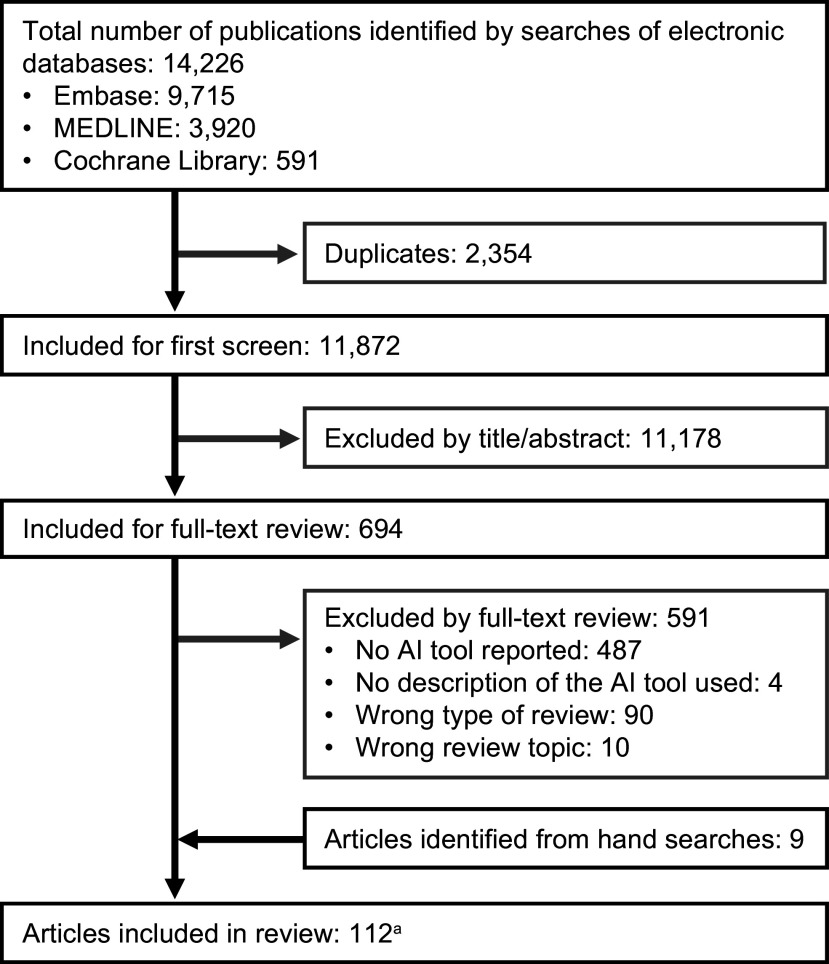
PRISMA flow diagram showing the results of the literature search.

In total, 14,226 records were retrieved from electronic literature database searches. After removing 2,354 duplicates, 11,872 records remained for title and abstract screening. The first round of screening excluded 11,178 records, leaving 694 full texts for review. Of these, 103 articles met the eligibility criteria, and an additional 9 eligible articles were identified through the bibliography searches, resulting in a total of 112 articles (Supplement B). These 112 articles represent 111 unique SLRs, as 2 articles reported different outcomes from the same SLR; information on AI tool use was extracted from only one of these. The PRISMA flowchart is shown in Figure 1.

### Health-related SLRs reporting on the use of AI tools

Among the 111 included studies (112 articles; Table 1), the majority were SLRs with narrative synthesis only (58 articles), followed by SLRs with meta-analysis (32 articles referring to 31 studies), scoping reviews (11 articles), rapid reviews (2 articles), umbrella reviews (2 articles), evidence gap maps (2 articles), an integrative review, a living review, a living review with meta-analysis, a rapid evidence mapping, and a systematic evidence mapping. The most common research areas covered by these reviews were neurology (15 articles), mental health (14 articles), health services research (14 articles referring to 13 studies), public health (10 articles), oncology (8 articles), cardiovascular health (8 articles), immunology (4 articles), and lifestyle research (4 articles).

**Table 1.** Characteristics of included studies

Reference^a^	Author affiliations	Type of review	Topic of review	AI tool	AI-assisted stage	Human-in-the-loop	Risk of bias
**Identified from database searches**							
Aali *et al.* 2020	Academic	Scoping review	Neurology	RobotReviewer	Risk of bias assessment	Yes	High
Agarwal *et al.* 2021	Academic	Systematic review	Health service	Cochrane RCT Classifier	Title/abstract screening	Yes	Low
Albuquerque *et al*. 2025	Academic	Systematic review	Gastroenterology	NLP toolkit (developed in Python)^b^	Search strategy development	No	High
Alfredo Ardisson Cirino Campos *et al.* 2025	Academic	Integrative review	Mental health	Rayyan	Title/abstract screening	Yes	Unclear
Al-Obeidat *et al*. 2024	Academic	Systematic review and meta-analysis	Oncology	Rayyan	Title/abstract screening	Yes	Low
Al-Sammarraie *et al*. 2025	Academic	Scoping review	Traditional medicine	GPT-4 Turbo	Data extraction	Yes	High
Anggreni *et al*. 2025	Academic	Systematic review	Endocrinology	NVivo	Data extraction	Yes	High
Aucoin *et al.* 2020	Academic	Scoping review	Mental health	Abstrackr	Title/abstract screening	Yes	High
Aujla *et al*. 2024	Academic	Systematic review and meta-analysis	Mental health	Nested Knowledge Auto, living semi-automated systematic review platform	Title/abstract screening	Yes	Low
Bagg *et al.* 2024	Academic	Systematic review	Neurology	Research Screener	Title/abstract screening	Yes	High
Balk *et al.* 2023a,b	Academic	Systematic review and meta-analysis	Health service	Abstrackr	Title/abstract screening	Yes	Low
Baron *et al.* 2013	Academic	Systematic review and meta-analysis	Gastroenterology	Automated text mining tool^b^	Title/abstract screening	Yes	High
Bell-Aldeghi *et al.* 2023	Academic	Systematic review	Health service	Automatic text classification algorithm (developed in Python)^b^	Title/abstract screening	Yes	High
Bilal *et al.* 2018	Academic	Systematic review and meta-analysis	Immunology	RobotReviewer	Risk of bias assessment	Unclear	High
Briand *et al.* 2025	Academic	Systematic review (state-of-the-art literature review)	Mental health	NVivo	Data extraction	Yes	Unclear
Brons et al., 2024	Academic	Scoping review	Public health	ASReview	Title/abstract screening	Yes	High
Brozek *et al.* 2024	Academic	Systematic review	Immunology	Laser AI	Data extraction	Yes	Low
Buchlak *et al.* 2022	Academic	Systematic review	Orthopedics	NLP analysis (developed in Python)^b^	Title/abstract screening	Yes	High
Buchlak *et al.* 2021	Academic	Systematic review	Neurology	NLP analysis (developed in Python)^b^	Title/abstract screening	Yes	High
Buchlak *et al.* 2020	Academic	Systematic review	Neurology	NLP analysis (developed in Python)^b^	Title/abstract screening	Yes	High
Burger *et al*. 2025	Academic	Systematic review and meta-analysis	Mental health	ASReview	Title/abstract screening	Yes	Low
Canova *et al*. 2024	Academic	Systematic review	Public health	ASReview	Title/abstract screening	Yes	Low
Carlson *et al.* 2023	Academic/Government	Systematic evidence mapping	Environmental health	DoCTER (ML and NLP)^b^	Title/abstract screening	Yes	High
				SWIFT-Active Screener	Title/abstract screening	Yes	
Chamseddine *et al.* 2022	Academic	Systematic review	Genetics	Semi-automated NLP algorithm^b^	Title/abstract screening	Yes	High
de Gans *et al.* 2024	Academic	Systematic review	Neurology	ASReview	Title/abstract screening	Yes	Low
da Silva *et al.* 2025	Academic	Systematic review	Neurology	Rayyan	Title/abstract screening	Yes	Low
da Silva Mulder *et al.* 2024	Academic/Government	Evidence gap map	Health service	Rayyan	Title/abstract screening	Yes	Low
Das *et al.* 2024	Academic	Systematic review and meta-analysis	Neurology	Research Screener	Title/abstract screening	Yes	Low
De Zwart & Ruis, 2024	Academic	Systematic review	Neurology	ASReview	Title/abstract screening	Yes	High
Demirtas Yilmaz. 2025	Academic	Systematic review	Audiology	Rayyan	Title/abstract screening	Yes	High
Ducreux *et al.* 2025	Academic	Systematic review	Genetics	ASReview	Title/abstract screening	Yes	Low
Feng Q *et al.* 2025	Academic/Industry	Systematic review	Obstetrics	Grobid - semi-automated workflow	Data extraction	Yes	Unclear
Feng X *et al.* 2025	Academic	Systematic review and meta-analysis	Cardiovascular health	BioMedGPT-LM-7B	Search strategy development	Yes	Low
					Risk of bias assessment	Yes	
Franzoi *et al.* 2024	Academic	Systematic review	Mental health	ASReview	Title/abstract screening	Yes	High
García-Torres *et al.* 2024	Academic	Systematic review	Health service	ChatGPT-4 and 4o	Data extraction	Yes	Low
Ghozy *et al.* 2024	Academic	Systematic review and meta-analysis	Neurology	AutoLit (Nested Knowledge)	Title/abstract screening	Yes	Low
					Data extraction	Yes	
Glenn *et al.* 2025	Academic	Systematic review	Public health	ASReview	Title/abstract screening	Yes	Low
Goldkuhle *et al.* 2018	Academic	Rapid review	Oncology	RobotReviewer	Data extraction	Yes	Low
					Risk of bias assessment		
Grinzinger *et al.* 2025	Academic	Systematic review	Oncology	scite.ai, consensus.ai.	Search strategy development	Yes	High
Halamoda‐Kenzaoui *et al.* 2022	Academic	Systematic review/feasibility study	Environmental health	SWIFT-Review	Title/abstract screening	Yes	High
				segmenteR (developed in R)^b^	Full-text screening	No	
				Syntactic parsing tool^b^	Full-text screening	No	
					Data extraction	No	
Hollands *et al.* 2019	Academic	Systematic review and meta-analysis	Lifestyle	EPPI-Reviewer 4	Title/abstract screening	Yes	Low
Hosseini *et al.* 2023	Academic	Systematic review	Genetics	Semi-automated NLP algorithm^b^	Title/abstract screening	Yes	High
Hsieh *et al.* 2024	Academic/Government	Systematic review	Neurology	DistillerSR	Title/abstract screening	Yes	Low
Iaconisi *et al.* 2024	Academic	Systematic review	Oncology	MySLR (Latent Dirichlet Allocation [LDA] algorithm)	Search strategy development	Yes	High
					Screening	Yes	
					Data extraction	Yes	
Jackson *et al.* 2022	Academic	Systematic review and meta-analysis	Lifestyle	An automated search strategy developed as part of the Human Behaviour Change Project (ML and NLP)^b^	Search strategy development	Yes	Low
Jayasundara *et al.* 2024	Academic	Systematic review and meta-analysis	Obstetrics	Rayyan	Title/abstract screening	Yes	High
Jayawardane *et al.* 2025	Academic/Government	Systematic review	Obstetrics	Rayyan	Title/abstract screening	Yes	High
Kang *et al*. 2025	Academic	Systematic review and meta-analysis	Dentistry	Rayyan	Title/abstract screening	Yes	Low
Karagiannis *et al.* 2019	Academic	Rapid review	Endocrinology	EPPI-Reviewer 4	Title/abstract screening	Yes	High
Kendall *et al.* 2025	Academic	Systematic review	Neurology	AutoLit (Nested Knowledge)	Data extraction	Yes	Low
Kim & Cruz, 2022	Academic	Systematic review and meta-analysis	Mental health	Leximancer	Data synthesis	No	High
Kumar *et al.* 2025	Academic	Systematic review and meta-analysis	Neuro-oncology	AutoLit (Nested Knowledge)	Search strategy development	Yes	Low
					Title/abstract screening	Yes	
					Data extraction	Yes	
Lam *et al.* 2019	Academic/ Government	Rapid evidence mapping	Lifestyle	SWIFT-Active Screener	Title/abstract screening	Yes	High
				SWIFT-Review	Data extraction	Yes	
Landau *et al*. 2024	Academic	Systematic review	Health service	ChatGPT 3.5	Data extraction	Yes	Unclear
Liu *et al.* 2025	Academic	Systematic review and meta-analysis	Athletic performance	ASReview	Title/abstract screening	Yes	Low
Lowe *et al.* 2021	Academic	Systematic review	Health service	Cochrane RCT Classifier	Title/abstract screening	Yes	Low
Marin *et al.* 2024	Academic	Umbrella review	Respiratory medicine	Nested Knowledge, SciSpace, Elicit, Perplexity	Data extraction	Yes	Low
McOwiti *et al.* 2024	Academic	Systematic review	Health service	ASReview	Title/abstract screening	Yes	High
Melinte *et al.* 2025	Academic	Systematic review and meta-analysis	Orthopedics	Research Rabbitt	Supplementary searches (citation searching)	Yes	Low
Meherali *et al.* 2025	Academic	Evidence Gap Map	Climate change	EPPI reviewer	Data extraction	Yes	High
Merino-Barbancho *et al.* 2025	Academic/Industry	Scoping review	Public health	ASReview	Title/abstract screening	Yes	High
Miranda *et al.* 2021	Academic	Systematic review	Mental health	ASReview	Title/abstract screening	Yes	High
Mushcab *et al.* 2025	Academic	Systematic review	Cardio-oncology	Elicit	Supplementary searches	Yes	Low
Napolitano *et al.* 2022	Academic	Systematic review	COVID-19	Topic modeling tool (developed in R)^b^	Title/abstract screening	Yes	High
				Semantic Scholar	Title/abstract screening		
				ML framework^b^	Title/abstract screening		
Nogueira *et al.* 2025	Academic	Systematic review	Aesthetic plastic surgery	Rayyan	Title/abstract screening	Yes	Low
Noteboom *et al.* 2024	Academic	Systematic review	Mental health	ASReview	Title/abstract screening	Yes	Low
Olaya-Mira *et al.* 2025	Academic	Systematic review	Prosthetics	Rayyan	Title/abstract screening	Yes	Unclear
Petrolini-Mateus *et al.* 2025	Academic	Systematic review	Public health	ChatGPT 4	Title/abstract screening	No	Low
Pillay *et al.* 2022	Academic	Living review	COVID-19	DistillerSR (Daisy AI)	Title/abstract screening	Yes	High
Pinna *et al.* 2020	Academic	Systematic review	Mental health	Rayyan	Title/abstract screening	Yes	High
Rakhshandehroo *et al.* 2023	Academic	Systematic review	Mental health	ASReview	Title/abstract screening	Yes	Unclear
Riaz *et al.* 2021	Academic	Living review and meta-analysis	Oncology	LIvE platform^b^	Citation screening (sub-stage unclear)	Yes	High
					Data synthesis	Unclear	
Robinson *et al.* 2024	Academic	Scoping review	Neurology	ChatGPT 4 (Bing chat)	Search strategy development	Yes	High
Sarbout *et al.* 2025	Academic	Systematic review	Ophthalmology	Elicit	Search strategy development	Yes	High
Shakeri Hossein Abad *et al.* 2021	Academic	Scoping review	Public health	NLP tools^b^	Search strategy development	Yes	High
Shemilt *et al.* 2013	Academic	Scoping review	Lifestyle	EPPI-Reviewer 4	Title/abstract screening	Yes	Low
Silva *et al.* 2022	Academic	Systematic review	Cardiovascular health	ASReview	Title/abstract screening	Yes	High
Slebe *et al.* 2024	Academic	Systematic review and meta-analysis	Endocrinology	ASReview	Title/abstract screening	Yes	Low
Sorrentino *et al.* 2024a	Academic	Systematic review	Public health	Rayyan	Title/abstract screening	Yes	Low
Sorrentino *et al.* 2024b	Academic	Systematic review	Public health	Rayyan	Title/abstract screening	Yes	Low
Spinelli *et al.* 2023	Academic	Systematic review	Gastroenterology	Rayyan	Title/abstract screening	Yes	High
Steele *et al.* 2025	Academic	Systematic review and meta-analysis	Mental health	Abstrackr	Title/abstract screening	Yes	Low
Sun *et al*. 2025	Academic	Scoping review	Respiratory medicine	ASReview	Title/abstract screening	Yes	Low
Susai *et al.* 2024	Academic	Systematic review	Public health	CADIMA	Title/abstract screening	Yes	High
Talukdar et al., 2024	Academic/Government	Systematic review and meta-analysis	Nephrology	Litmaps	Supplementary searches (snowballling)	No	Low
Teperikidis *et al.* 2023	Academic	Umbrella review	Cardiovascular health	ChatGPT	Search strategy development	Yes	High
					Title/abstract screening	Yes	
					Data extraction	Yes	
					Risk of bias assessment	Yes	
					Data synthesis	Yes	
Tun *et al.* 2025	Academic	Systematic review	Health service	Elicit	Data extraction	Yes	High
Valizadeh *et al.* 2025	Academic	Systematic review and meta-analysis	Oncology	AutoLit (Nested Knowledge)	Title/abstract screening	Yes	Low
					Data extraction	Yes	
Valizadeh *et al.* 2025	Academic	Systematic review and meta-analysis	Oncology	AutoLit (Nested Knowledge)	Title/abstract screening	Yes	Low
					Data extraction	Yes	
Vallury *et al.* 2015	Academic	Systematic review	Mental health	Nvivo	Full-text screening	Yes	Unclear
van den Berg et al., 2025	Academic	Scoping review	Neurology	ASReview	Title/abstract screening	Yes	Low
Van Dijk *et al.* 2024	Academic	Systematic review	Cardiovascular health	ASReview	Title/abstract screening	Yes	Low
Visser *et al.* 2025	Academic	Systematic review	Health service	ASReview	Title/abstract screening	Yes	Low
Vizcarra *et al.* 2024	Academic	Systematic review	Neurology	Python and ChatGPT-4	Title/abstract screening	Yes	Unclear
Voorn *et al.* 2025	Academic	Systematic review and meta-analysis	Geriatrics	ASReview	Title/abstract screening	Yes	Low
Wen *et al.* 2025	Academic	Systematic review and meta-analysis	Hepatology	ASReview	Title/abstract screening	Yes	Low
Westendorp *et al.* 2023	Academic	Systematic review	Health service	ASReview	Title/abstract screening	Yes	Low
Yamikan *et al.* 2025	Academic	Systematic review	Cardiovascular health	Rayyan	Title/abstract screening	Yes	Low
Yappalparvi *et al.* 2025	Academic	Systematic review and meta-analysis	Cardiovascular health	AutoLit (Nested Knowledge)	Title/abstract screening	Yes	High
					Data extraction	Yes	
Yazicioglu *et al.* 2025	Academic	Systematic review	Health service	Rayyan	Title/abstract screening	Yes	Low
Zamantakis *et al.* 2024	Academic	Systematic review	Public health	Semi-automated text mining and natural language processing (custom)	Title/abstract screening	No	Unclear
Zhu *et al.* 2023	Academic	Scoping review	Health service	ASReview	Title/abstract screening	Yes	High
**Identified from hand searches**							
Crossingham *et al.* 2021	Academic/ Government	Systematic review and meta-analysis	Immunology	Cochrane RCT Classifier	Title/abstract screening	Yes	Low
Eun *et al.* 2021	Academic	Systematic review and meta-analysis	Cardiovascular health	ML approach (developed in Python)^b^	Title/abstract screening	Yes	High
Foulquier *et al.* 2018	Academic	Systematic review	Immunology	BIBOT (NLP; developed in Python)^b^	Title/abstract screening	No	High
Gaskins *et al.* 2021	Academic	Systematic review	Neurology	Rayyan	Title/abstract screening	Yes	High
Giummarra *et al.* 2020	Academic	Systematic review	Mental health	Abstrackr	Title/abstract screening	Yes	Low
				Wordstat and QDA Minder	Full-text screening	Yes	
Rogers *et al.* 2020	Academic	Systematic review and meta-analysis	Oncology	Rayyan	Title/abstract screening	Yes	Low
Viner *et al.* 2022	Academic	Systematic review	COVID-19	EPPI-Reviewer 4	Title/abstract screening	Yes	High
Xiong *et al.* 2018	Academic	Systematic review and meta-analysis	Cardiovascular health	ML approach (developed in R)^b^	Title/abstract screening	Yes	High
Yamamoto *et al.* 2021	Academic	Systematic review and meta-analysis	Nephrology	RobotAnalyst	Title/abstract screening	Yes	High

a The articles identified in the systematic literature review are listed in Supplement B.

b Custom tools.

AI, artificial intelligence; ML, machine learning; NLP, natural language processing; RCT, randomized controlled trial.

**Figure 2.**
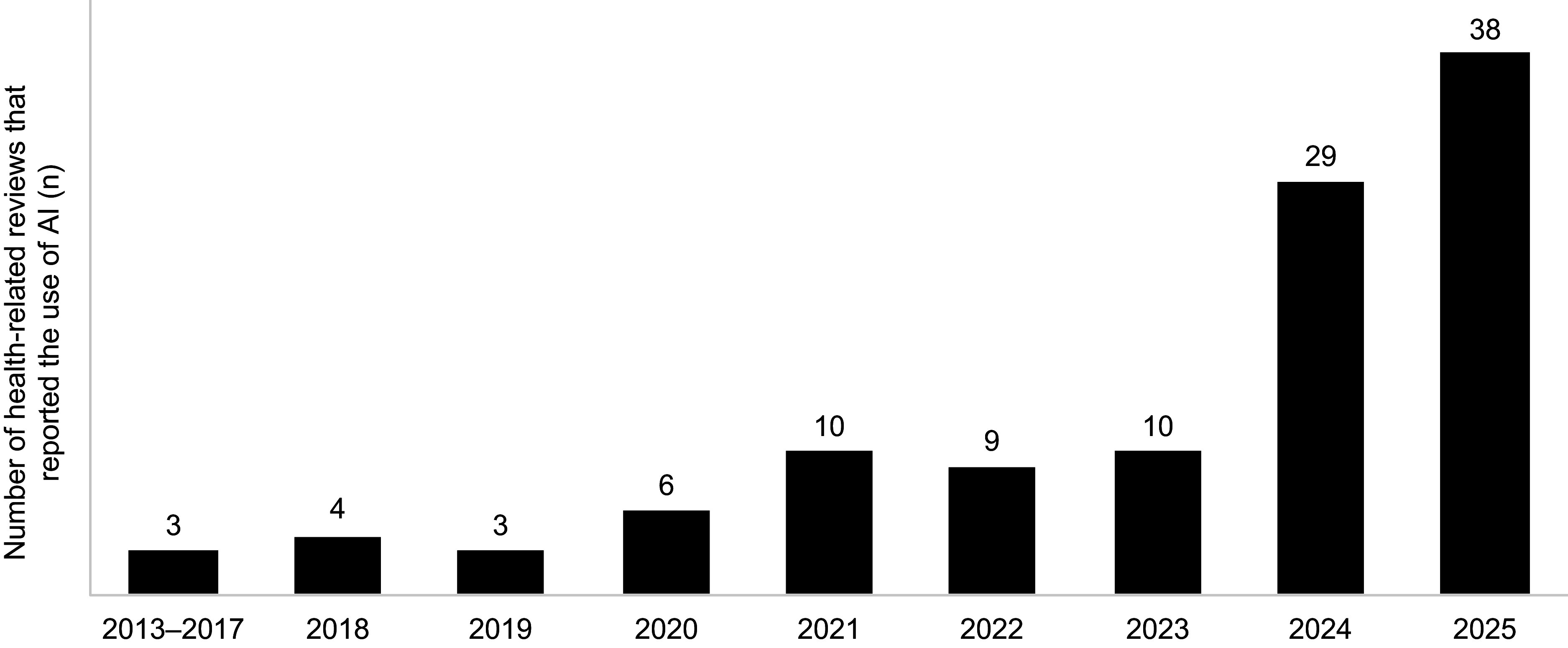
Number of health-related reviews reporting on the use of AI over the years.

The earliest reviews reporting the use of AI tools were published in 2013, with an increasing number of AI-assisted reviews identified over time (Figure 2). Among the 112 included articles (111 unique SLRs), 91.1 percent were published since 2020. For 2025, 38 AI-assisted SLRs had been published as of September 2025. Of the 112 articles, 103 were authored solely by researchers from academic institutions, 7 involved collaborations between academia and government organizations, and 2 were collaborations between academia and the pharmaceutical industry (Table 1).

Although the findings from the included reviews were not synthesized in the current study, the ROBIS tool was used to assess the quality of AI-assisted reviews. Among the 111 included studies, 51 (45.9 percent) had a high risk of bias, 51 studies (45.9 percent) had a low risk of bias, and 9 studies (8.1 percent) had an unclear risk of bias (Supplementary Table 3).

### Adoption of AI in SLRs and usage trends

In total, 134 implementations of AI were identified across the 111 included reviews. Within these, forty-five distinct AI tools were used, comprising twenty-nine publicly available software-based tools and sixteen custom tools developed by the article authors.

**Figure 3.**
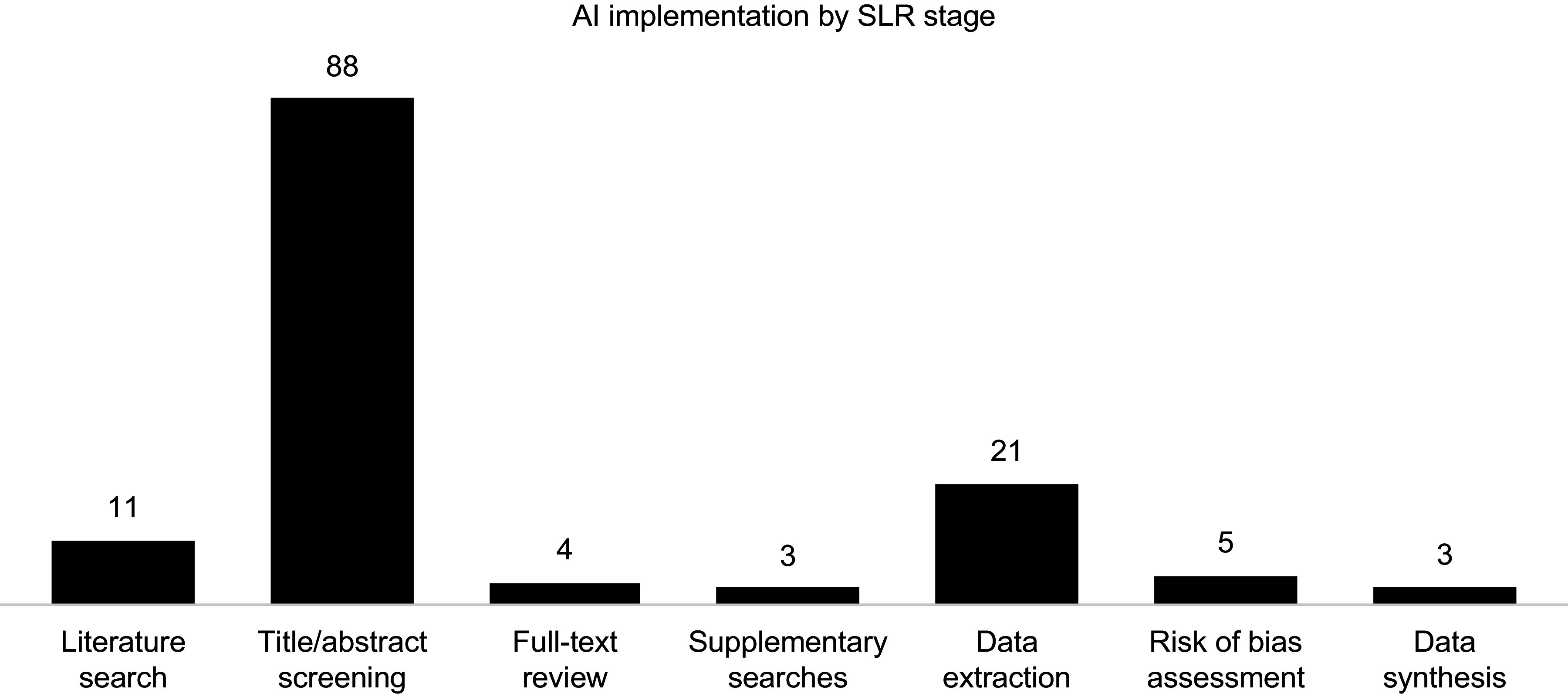
Use of AI software across the different stages of an SLR.

AI tools were used most frequently for title and abstract screening (eighty-eight instances, including two studies in which the screening stage was unspecified), followed by data extraction (twenty-one instances), search strategy development (ten instances), risk-of-bias assessment (five instances), full-text review (four instances), data synthesis (three instances), and supplementary searches (three instances) (Figure 3).

**Figure 4.**
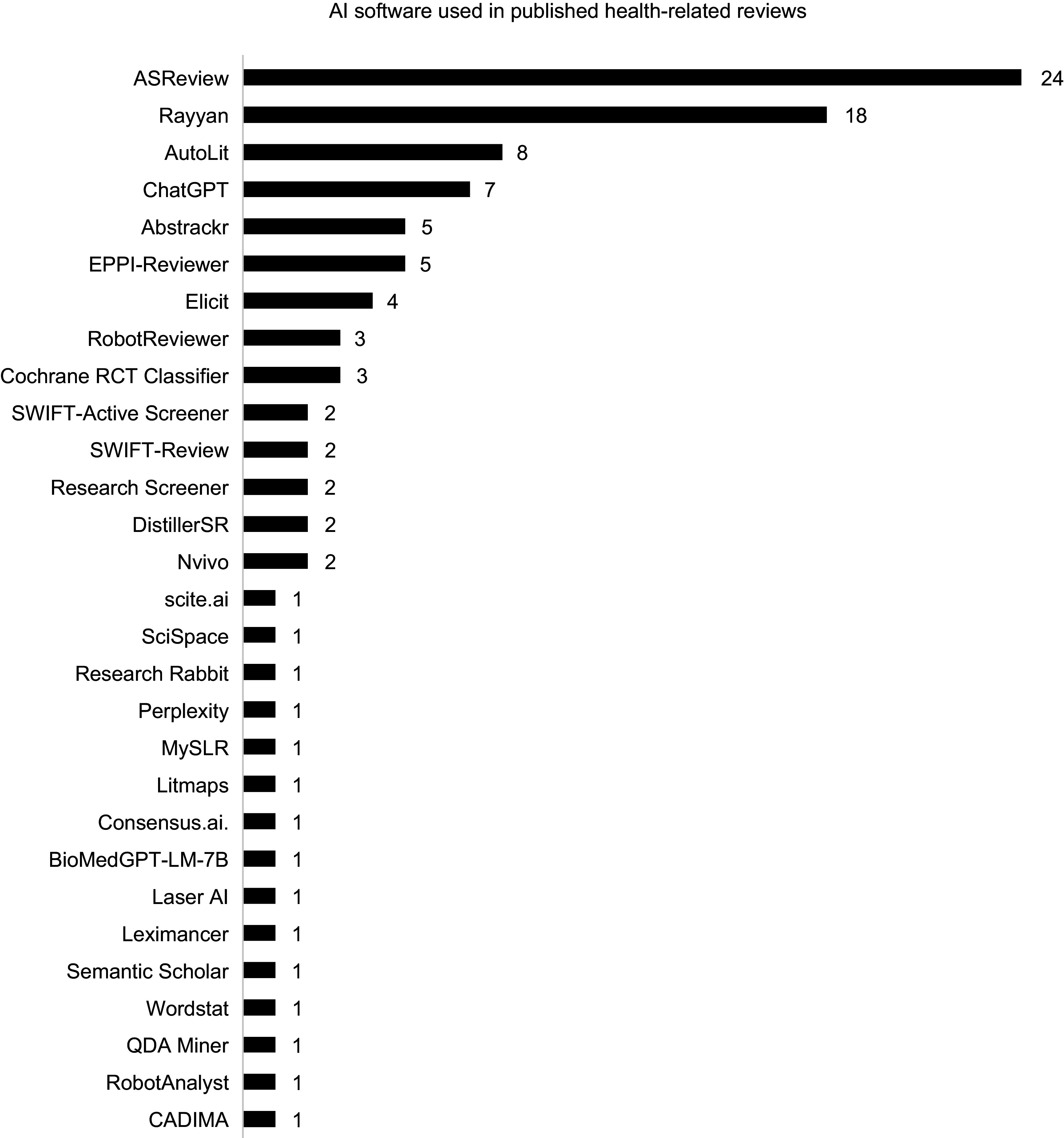
AI software used in published health-related reviews.

The most frequently used AI software was ASReview (twenty-four reviews), followed by the ML functionality in Rayyan (eighteen reviews), Autolit (Nested Knowledge; eight reviews) and EPPI-Reviewer (five reviews), all of which were used for title and abstract screening (EPPI-Reviewer was also used for data extraction and AutoLit was also used for data extraction and search strategy development). In addition to tools specifically designed for SLRs, OpenAI’s generative AI models (such as ChatGPT and other GPT-based systems) were used for multiple tasks in eight separate reviews, including search strategy generation, title and abstract screening, data extraction, risk-of-bias assessment, data synthesis, and report writing. The remaining AI software tools were each used in five or fewer studies (Figure 4). Although some authors who used custom tools shared their code, many of these tools are likely to be inaccessible to the public.

#### Search strategy development

AI tools were used for search strategy development in twelve instances. Three studies applied LLMs (two using ChatGPT and one using BioMedGPT-LM-7B) and five studies employed platform-based tools such as Elicit, Nested Knowledge, MySLR, and scite.ai/consensus.ai to identify relevant concepts and expand terminology. The remaining three studies used custom NLP- or ML-based tools, including an automated search strategy developed as part of the Human Behaviour Change Project.

#### Title/abstract screening

Title and abstract screening was the most common stage for use of AI tools, with eighty-eight instances, including two studies in which the screening stage (title/abstract or full text) was unspecified. Various software-based tools were used for screening, with ASReview being the most frequently used (twenty-four instances). The primary AI feature applied during title and abstract screening was active learning–based relevance prediction, in which the algorithm continuously reprioritized records based on reviewer feedback to optimize efficiency and accuracy. Similarly, most custom tools were developed for screening. The main features of these tools included relevance prediction and topic modeling. Some custom tools were also designed to calculate scores based on metrics, such as the presence and frequency of keywords, to determine study relevance.

#### Full-text screening

AI-assisted full-text screening was identified in four instances. In total, three software-based text analysis tools – Nvivo, Wordstat, and QDA Miner – were used to search for keywords and synonyms indicating relevance to the research question. In one review, the authors developed a custom article segmentation tool that extracted the methods and results sections from full-text articles. A syntactic parsing tool was then applied to identify studies containing predefined keywords, enabling full automation of citation screening in that study.

#### Supplementary searches

AI was used for supplementary searches in three instances, with Research Rabbit, Elicit, and with Litmaps. In the SLRs that used Research Rabbit and Litmaps, the AI features of these tools were applied to identify articles related to those retrieved through the main database searches. When Elicit was used, it employed the same search terms as those used in the main database searches.

#### Data extraction

AI was used for data extraction in twenty-one instances, with AutoLit (Nested Knowledge) and GPT-based models being the most frequently employed (seven and four instances, respectively). The most utilized AI feature was NLP–based automated data extraction, in which LLMs parsed full-text articles to identify and populate structured variables such as study characteristics, interventions, and outcomes. In one study, a custom syntactic parsing tool used for full-text screening was also applied in data extraction to collect information related to the research question.

#### Risk-of-bias assessment

AI was used for risk-of-bias assessment in five instances. In three studies, RobotReviewer, a software-based tool, was used to assess risk of bias in RCTs. In another study, ChatGPT was utilized with prompts based on AMSTAR 2.0, a risk-of-bias assessment tool for systematic reviews. In the fifth study, BioMedGPT-LM-7B, a generative AI model trained on Cochrane-rated RCTs, was used to automatically assess risk of bias.

#### Data synthesis

AI was used for data synthesis in three instances. In one study, Leximancer, a software-based tool, was used for qualitative (thematic) data synthesis. Another software-based tool, an ML-based RCT classifier, was integrated into a living review platform (LIvE) and applied during the data synthesis step. In the third study, ChatGPT was explored for data synthesis and report writing.

### Functionality of current AI tools

In the identified studies, none of the AI tools operated fully autonomously. Human reviewers played a critical role at all SLR stages, with AI tools primarily serving as support systems. (Supplementary Table 4). For title and abstract screening, most software-based AI tools required training with manually labeled records before implementation. Following this, priority screening was applied, for which the most relevant records were prioritized for human reviewers to assess. After this step, either all remaining records were manually screened or the authors defined a stopping rule (i.e., the number of consecutive exclusions required to stop manual screening). For data extraction and risk-of-bias assessment, human reviewers were involved in complementing or verifying extracted data and assessment results. However, thematic data synthesis with Leximancer was conducted without human intervention. Custom AI workflows also required human participation at various stages, including training, manual screening, and verification, similarly to software-based tools.

### Adoption of AI in HTAs

As of October 2025, few HTA agencies had issued formal guidance on the use of AI tools for SLRs. Only the Institute for Quality and Efficiency in Health Care (IQWiG) in Germany (17), the National Institute for Health and Care Excellence (NICE) in the United Kingdom (18), and Canada’s Drug Agency (CDA-AMC) (15)) have published official guidance on the potential application of AI or ML methods in the evidence-generation process (Supplementary Table 5). In 2019, EUnetHTA published a detailed guideline for information retrieval for SLRs and HTAs that mentioned early references to text-analytic approaches for search-term identification (16). The JBI Manual for Evidence Synthesis and the Cochrane Handbook have included recommendations on the use of AI at different stages of the review process (23).

#### Search strategy development

IQWiG explicitly supports the use of text analysis and ML methods to aid in developing search strategies (17), and NICE’s position statement (18) acknowledges that AI and LLMs can assist with search strategy generation. EUnetHTA’s information retrieval guideline also refers to text-analytic approaches for identifying search terms (16). Similarly, the Cochrane Handbook recommends text-mining and frequency-analysis techniques to identify relevant terms and concepts (23).

#### Citation screening

IQWiG’s latest General Methods note that validated classifiers and other ML approaches may assist in title and abstract screening (17), whereas NICE recognizes that AI tools can help to classify study designs and prioritize records for review (18). EUnetHTA refers to screening platforms such as Covidence, Rayyan, and EPPI-Reviewer that can facilitate reviewer collaboration and documenting the screening process, although it does not provide AI-specific recommendations (16). The Cochrane Handbook explicitly describes the Cochrane RCT Classifier and Cochrane Crowd as validated tools that support semiautomated RCT identification and highlights the value of ML and text-mining to streamline screening workflows (23).

#### Data extraction and risk risk-of of-bias assessment

The Cochrane Handbook discusses the potential use of NLP and ML tools to automate data extraction and risk-of-bias assessment but does not recommend specific systems owing to their limited validation at the time of publication (23). IQWiG, NICE, and EUnetHTA have not yet provided formal recommendations on AI-assisted data extraction or risk-of-bias assessment in their methodological guidance.

### Advantages and disadvantages of AI tools

One key advantage of AI tools for title/abstract screening, data extraction, and risk-of-bias assessment was substantial time savings (Supplementary Table 6). AI-assisted screening reduced the manual workload by limiting the number of studies requiring human review, particularly during citation screening. Reviewers also noted that AI tools generally produced accurate outputs and improved reviewer performance by increasing objectivity.

However, limitations were also noted. Some studies reported AI errors and raised concerns that relevant records might be missed during screening. Reviews also highlighted the absence of validated stopping rules for priority screening, increasing the risk of omitting eligible studies if manual screening stopped too early. Rank-order bias was another concern, as the ranking of records could influence inclusion decisions, although some reviews suggested that alternative validation approaches may help to mitigate this risk.

## Discussion

To the authors’ knowledge, this study is the first to systematically assess the use of AI tools in healthcare-related SLRs, addressing three key objectives: (i) quantifying published SLRs reporting use of AI tools; (ii) identifying AI tools used at each SLR stage and their functionalities; and (iii) assessing whether HTA agencies accept or recommend AI tools. Across 111 identified SLRs, 45 distinct AI tools were reported, most frequently used for title and abstract screening. Human oversight remains critical, as no studies used fully autonomous AI. Few HTA agencies have issued formal guidance on AI in SLRs (15–18) suggesting limited current acceptance for HTA decision making. These findings highlight the growing role of AI in evidence synthesis and the need for further evaluation of its integration into HTA processes. Notably, the use of AI tools in SLRs consistently incorporated human oversight, reflecting current HTA positions that automation should support, rather than replace, human expert judgment.

AI appeared in SLRs in 2013, with over 90 percent of AI-assisted reviews published since 2020. This trend may reflect the influence of PRISMA 2020 guidance, which introduced requirements to report automation tools in study selection and data-collection processes (22). Among the 111 included reviews, AI was mostly used in the title and abstract screening stage (88 instances, including 2 studies in which the screening stage was unspecified), but fewer studies applied AI for data extraction (21 instances), full-text review (4 instances), risk-of-bias assessment (5 instances), literature searches (10 instances), data synthesis (3 instances), and supplementary searches (3 instances). In total, twenty-nine publicly available software-based tools and sixteen custom-built tools were identified. However, reporting inconsistencies and indexing limitations likely mean these studies represent a fraction of healthcare-related SLRs, and some tools may also be used internally without disclosure. Insufficient guidance on AI use in systematic reviews may hinder transparent reporting, as evidenced by inconsistencies observed in peer-reviewed publications. For HTA processes, a lack of clarity in this regard could limit reproducibility and confidence in evidence generation, underscoring the need for clear standards. Emerging guidelines, such as ELEVATE-GenAI for reporting LLM use in Health Economics and Outcomes Research (27), highlight growing interest in standardizing AI reporting in health-related research. Most reviews were conducted by academic or research institutions, with only a small proportion involving collaborations with government or industry partners. This indicates that AI applications in systematic reviews remain largely driven by the academic research community.

AI can automate repetitive and time-consuming SLR tasks, allowing researchers to focus on higher-level tasks. Most AI tools employed a human-in-the-loop approach, supporting rather than replacing human reviewers. Although generative AI and agent-based systems are advancing, none of the identified studies used fully autonomous AI, consistent with HTA expectations (18). Reported limitations included potential errors, missed studies, and data reliability concerns, reinforcing the need for human oversight. These findings align with previous research on AI use highlighting challenges related to data validity, transparency, and systemic biases (28;29). Nonetheless, AI offers benefits in terms of efficiency and consistency by reducing variability in subjective judgment (11), identifying patterns and insights that may not be immediately apparent to human reviewers, and minimizing human bias and fatigue. Overall, when used to complement human expertise in SLRs, AI should enable faster high-quality reviews that ultimately benefit HTA decision making (11;30).

Although CDA-AMC, IQWiG, NICE, EUnetHTA, JBI and Cochrane have recommended several AI functionalities for SLRs (15–18), formal HTA guidelines remain limited. Barriers to adoption of AI in HTA include data quality concerns, limited technical expertise, infrastructure constraints, regulatory uncertainty, and ethical or policy issues (28;29).

The future of AI in SLRs and HTAs is likely to expand with advancements in AI technologies and the development of LLMs. Although current AI tools are mainly designed for title and abstract screening, future developments will likely assist with full-text review, data extraction, and risk-of-bias assessment. Generative AI can already summarize large volumes of text, generate insights, and draft sections of reports (31), supporting dynamic methodologies such as in living reviews and living HTA. AI can also manage increasing workloads driven by external circumstances. For instance, the implementation of the Joint Clinical Assessment process in January 2025 (7;19;20) may drive increased AI involvement in HTA-related evidence synthesis, particularly in identifying PICO criteria and conducting indirect treatment comparisons. The integration of AI into SLRs for HTA could ultimately accelerate timelines and expedite patient access to treatments.

The findings of this comprehensive assessment of AI use in health-related SLR publications build on prior research, such as Blaizot *et al.* (21), which examined a similar topic but covered a narrower scope. Compared with Blaizot *et al.*, this study identified a greater number of relevant articles, not only because of a later search date but also because of the broader inclusion of AI applications beyond screening. This study also extends a review by Khalil *et al.* (32) by incorporating the emerging generative-AI period and evaluating their relevance to HTA guidance and practice.

Several limitations should be noted. First, a single reviewer conducted the screening process, increasing the risk of bias. However, this limitation was partially mitigated by adhering to standard SLR methodology and by validating included studies against prior SLRs. Second, the literature search was limited to English-language publications, which may have excluded some relevant studies; however, our comprehensive search of HTA websites, including fifty-three members of INAHTA and EUnetHTA, would have ensured that the key international guidance on AI use in SLRs was captured regardless of the primary language of publication. Third, only studies that explicitly mentioned AI in the title or abstract were included for full-text review, potentially excluding eligible studies that referenced AI use only in the full-text. However, this is a standard limitation of SLRs because title and abstract screening is a widely accepted approach for managing large search results. Fourth, half of the included studies had a high or unclear risk of bias as measured by the ROBIS tool. However, this was expected because ROBIS requires at least two human reviewers for screening, data extraction, and risk-of-bias assessment. Yet, in many cases, AI tools replaced one or more human reviewers, and the extent of human involvement in these stages was often unclear. Finally, given the rapid evolution of AI tools, it is also possible that new AI-assisted SLRs have been conducted since September 2025, when the searches were completed.

## Conclusions

This study found that AI was reported as used in 112 articles about 111 unique health and medical research SLRs. Although commercial and custom tools now support all stages of SLR development, most commonly title and abstract screening, the adoption rate remains low, and human involvement remains critical. AI use in SLRs is increasing, with over 90 percent of AI-assisted reviews identified published since 2020. Few HTA agencies have provided guidelines on the use of AI tools for SLRs, suggesting limited current acceptability in HTA decision making. As AI technologies evolve, further research is needed to assess performance and reliability to inform guideline updates. Collaborative consensus exercises involving HTA agencies and methodological experts could also help to refine existing guidance and develop standardized criteria for validating AI tools in SLRs. These developments will contribute to more timely and efficient healthcare decision making, potentially accelerating patient access to treatments.

## Supporting information

10.1017/S0266462326103535.sm001 Abogunrin et al. supplementary material Abogunrin et al. supplementary material

## Supplementary material

The supplementary material for this article can be found at http://doi.org/10.1017/S0266462326103535.
