# Supplementary material for: A systematic literature review (SLR) on the adoption of artificial intelligence-assisted SLRS: implications for health technology assessments
Source: Int J Technol Assess Health Care. 2026 Feb 16;42(1):e29. doi: 10.1017/S0266462326103535 (PMC13071852; doi:10.1017/S0266462326103535)
Supplement: Abogunrin et al. supplementary material [file S0266462326103535sup001.docx]

# Supplementary materials

## Supplement A. Risk of bias assessment

The Risk Of Bias In Systematic Reviews (ROBIS) tool evaluates four domains for risk of bias in a systematic review, each with five or six signaling questions (22). Questions were answered Yes (Y), Probably Yes (PY), Probably No (PN), No (N), or No Information (NI). If any of the signaling questions were answered N or PN, then the concern for this domain is high. If all questions were answered Y or PY, then the concern is low. If there were questions that were answered NI and all other questions were Y or PY, then the concern for this domain is unclear.

1. Study eligibility criteria

1) Did the review adhere to pre-defined objectives and eligibility criteria?

2) Were the eligibility criteria appropriate for the review question?

3) Were eligibility criteria unambiguous?

4) Were any restrictions in eligibility criteria based on study characteristics appropriate (e.g., date, sample size, study quality, outcomes measured)?

5) Were any restrictions in eligibility criteria based on sources of information appropriate (e.g., publication status or format, language, availability of data)?

1. Identification and selection of studies

1) Did the search include an appropriate range of databases/electronic sources for published and unpublished reports?

2) Were methods additional to database searching used to identify relevant reports?

3) Were the terms and structure of the search strategy likely to retrieve as many eligible studies as possible?

4) Were restrictions based on date, publication format, or language appropriate?

5) Were efforts made to minimize error in selection of studies?

1. Data collection and study appraisal

1) Were efforts made to minimize error in data collection?

2) Were sufficient study characteristics available for both review authors and readers to be able to interpret the results?

3) Were all relevant study results collected for use in the synthesis?

4) Was risk of bias (or methodological quality) formally assessed using appropriate criteria?

5) Were efforts made to minimize error in risk of bias assessment?

1. Synthesis and findings

1) Did the synthesis include all studies that it should?

2) Were all pre-defined analyses reported or departures explained?

3) Was the synthesis appropriate given the nature and similarity in the research questions, study designs and outcomes across included studies?

4) Was between-study variation (heterogeneity) minimal or addressed in the synthesis?

5) Were the findings robust (e.g., as demonstrated through funnel plot or sensitivity analyses)?

7) Were biases in primary studies minimal or addressed in the synthesis?

After this evaluation, an overall judgment of the risk of bias was made using the following signaling questions. These questions mean that even if there were concerns identified in the four domains, the overall risk of bias could still be low if the impact of these concerns were discussed by the authors.

1. Did the interpretation of findings address all of the concerns identified in domains 1 to 4?
2. Was the relevance of identified studies to the review’s research question appropriately considered?
3. Did the reviewers avoid emphasizing results on the basis of their statistical significance?

## Supplement B. Articles included in the systematic literature review

1. **Aali G., Drummond A., das Nair R., Shokraneh F.** Post-stroke fatigue: a scoping review. *F1000Res*. **9**,242 (2020)

2. **Agarwal S., Glenton C., Tamrat T., Henschke N., Maayan N., Fonhus M.S.*, et al.*** Decision-support tools via mobile devices to improve quality of care in primary healthcare settings. *Cochrane Database Syst Rev*. **7**(7),CD012944 (2021)

3. **Al-Obeidat F., Hafez W., Gador M., Ahmed N., Abdeljawad M.M., Yadav A.*, et al.*** Diagnostic performance of AI-based models versus physicians among patients with hepatocellular carcinoma: a systematic review and meta-analysis. *Front*. **7**,1398205 (2024)

4. **Al-Sammarraie R.N., Al Mubasher H., Awad M., Naalbandian S., Darwiche N., Zurayk R.*, et al.*** An artificial intelligence-aided scoping review of medicinal plant research in the Fertile Crescent. *Frontiers in Pharmacology*. **16**(no pagination), (2025)

5. **Albuquerque C., Neves P.A., Godinho A., Zdravevski E., Lameski P., Pires I.M.*, et al.*** Colonoscopy image analysis for polyp detection: A systematic review of existing approaches and opportunities. *Intelligence Based Medicine*. **12**(no pagination), (2025)

6. **Alfredo Ardisson Cirino Campos F., Feitosa F.B., Moll M.F., Reis I.D.O., Sanchez Garcia J.C., Ventura C.A.A.** Initial Requirements for the Prototyping of an App for a Psychosocial Rehabilitation Project: An Integrative Review. *International Journal of Environmental Research and Public Health*. **22**(2), (2025)

7. **Anggreni N., Kristianto H., Handayani D., Yueniwati Y., Irawan P.L.T., Rosandi R.*, et al.*** Artificial Intelligence for Diabetic Foot Screening Based on Digital Image Analysis: A Systematic Review. *J Diabetes Sci Technol*.19322968251317521 (2025)

8. **Aucoin M., LaChance L., Cooley K., Kidd S.** Diet and psychosis: a scoping review. *Neuropsychobiology*. **79**(1),20-42 (2020)

9. **Aujla S., Sandeep M., Aparnavi P., Padhi B.K., Shamim M.A., Sahoo S.*, et al.*** Effect of maternal obsessive-compulsive disorder (OCD) on feto-maternal outcomes: A systematic review and meta-analysis. *International Journal of Gynecology and Obstetrics*. **167**(3),949-56 (2024)

10. **Bagg M.K., Hellewell S.C., Keeves J., Antonic-Baker A., McKimmie A., Hicks A.J.*, et al.*** The Australian Traumatic Brain Injury Initiative: Systematic Review of Predictive Value of Biological Markers for People With Moderate-Severe Traumatic Brain Injury. *J Neurotrauma*. (2024)

11. **Balk E.M., Danilack V.A., Bhuma M.R., Cao W., Adam G.P., Konnyu K.J.*, et al.*** Reduced Compared With Traditional Schedules for Routine Antenatal Visits: A Systematic Review. *Obstet Gynecol*. **142**(1),8-18 (2023)

12. **Balk E.M., Danilack V.A., Cao W., Bhuma M.R., Adam G.P., Konnyu K.J.*, et al.*** Televisits Compared With In-Person Visits for Routine Antenatal Care: A Systematic Review. *Obstet Gynecol*. **142**(1),19-29 (2023)

13. **Baron J.A., Senn S., Voelker M., Lanas A., Laurora I., Thielemann W.*, et al.*** Gastrointestinal adverse effects of short-term aspirin use: a meta-analysis of published randomized controlled trials. *Drugs R D*. **13**(1),9-16 (2013)

14. **Bell-Aldeghi R., Gibrat B., Rapp T., Chauvin P., Guern M.L., Billaudeau N.*, et al.*** Determinants of the Cost-Effectiveness of Telemedicine: Systematic Screening and Quantitative Analysis of the Literature. *Telemed J E Health*. **29**(7),1078-87 (2023)

15. **Bilal J., Riaz I.B., Kamal M.U., Elyan M., Sudano D., Khan M.A.** A Systematic Review and Meta-analysis of Efficacy and Safety of Novel Interleukin Inhibitors in the Management of Psoriatic Arthritis. *J Clin Rheumatol*. **24**(1),6-13 (2018)

16. **Briand C., Vallee C., Luconi F., Theriault J., Sauvageau A., Bellemare J.** State-of-the-art literature review of Recovery College evaluative studies between 2013-2024. *Frontiers in Psychiatry*. **16**(no pagination), (2025)

17. **Brons A., Wang S., Visser B., Krose B., Bakkes S., Veltkamp R.** Machine Learning Methods to Personalize Persuasive Strategies in mHealth Interventions That Promote Physical Activity: Scoping Review and Categorization Overview. *Journal of Medical Internet Research*. **26**(no pagination), (2024)

18. **Brozek J., Borowiack E., Sadowska E., Nowak A., Sousa-Pinto B., Vieira R.J.*, et al.*** Patients' values and preferences for health states in allergic rhinitis-An artificial intelligence supported systematic review. *Allergy*. **79**(7),1812-30 (2024)

19. **Buchlak Q.D., Clair J., Esmaili N., Barmare A., Chandrasekaran S.** Clinical outcomes associated with robotic and computer-navigated total knee arthroplasty: a machine learning-augmented systematic review. *Eur J Orthop Surg Traumatol*. **32**(5),915-31 (2022)

20. **Buchlak Q.D., Esmaili N., Leveque J.C., Bennett C., Farrokhi F., Piccardi M.** Machine learning applications to neuroimaging for glioma detection and classification: An artificial intelligence augmented systematic review. *J Clin Neurosci*. **89**,177-98 (2021)

21. **Buchlak Q.D., Esmaili N., Leveque J.C., Farrokhi F., Bennett C., Piccardi M.*, et al.*** Machine learning applications to clinical decision support in neurosurgery: an artificial intelligence augmented systematic review. *Neurosurg Rev*. **43**(5),1235-53 (2020)

22. **Burger P., Bos R.W., Maas J., Simeunovic-Ostojic M., Gemke R.J.B.J.** Sleep disturbances in anorexia nervosa. *European Eating Disorders Review*. **33**(2),318-42 (2025)

23. **Canova C., Dansero L., Destefanis C., Benna C., Rosato I.** Assessing the health status of migrants upon arrival in Europe: a systematic review of the adverse impact of migration journeys. *Globalization and Health*. **20**(1), (2024)

24. **Carlson L.M., Christensen K., Sagiv S.K., Rajan P., Klocke C.R., Lein P.J.*, et al.*** A systematic evidence map for the evaluation of noncancer health effects and exposures to polychlorinated biphenyl mixtures. *Environ Res*. **220**,115148 (2023)

25. **Chamseddine R.S., Wang C., Yin K., Wang J., Singh P., Zhou J.*, et al.*** Penetrance of male breast cancer susceptibility genes: a systematic review. *Breast Cancer Res Treat*. **191**(1),31-8 (2022)

26. **da Silva Mulder J.N., Ramos Pinto M., Anibal I., Dornellas A.P., Garrido D., Huanca C.*, et al.*** Teledentistry Applied to Health and Education Outcomes: Evidence Gap Map. *Journal of Medical Internet Research*. **26**(1), (2024)

27. **da Silva N.N., Cavalcante W.A., Tertuliano A.L.O., de Sousa A.A.A., de Oliveira D.C.A., Santos A.B.D.*, et al.*** Conductive Education for Children With Cerebral Palsy: A Systematic Review of Outcomes, Practice Time and Motor Performance Assessment. *Child: care, health and development*. **51**(5), (2025)

28. **Das N., Nguyen P., Ho T.Q.A., Lee P., Robinson S., Gao L.** Methods for Measuring and Valuing Informal Care: A Systematic Review and Meta-Analysis in Stroke. *Value Health*. **27**(12),1789-804 (2024)

29. **de Gans C.J., Burger P., van den Ende E.S., Hermanides J., Nanayakkara P.W.B., Gemke R.*, et al.*** Sleep assessment using EEG-based wearables - A systematic review. *Sleep Med Rev*. **76**,101951 (2024)

30. **de Zwart B., Ruis C.** An update on tests used for intraoperative monitoring of cognition during awake craniotomy. *Acta Neurochir (Wien)*. **166**(1),204 (2024)

31. **Demirtas Yilmaz B.** Prediction of Auditory Performance in Cochlear Implants Using Machine Learning Methods: A Systematic Review. *Audiology Research*. **15**(3), (2025)

32. **Ducreux B., Patrat C., Firmin J., Ferreux L., Chapron C., Marcellin L.*, et al.*** Systematic review on the DNA methylation role in endometriosis: current evidence and perspectives. *Clinical Epigenetics*. **17**(1), (2025)

33. **Feng Q., Crispin J., Longobardi S., D'Hooghe T., Mol B.W., Li W.** Trial characteristics, geographic distribution, and selected methodological issues of 1425 infertility trials published from 2012 to 2023: a systematic review. *Human Reproduction Open*. (pagination), (2025)

34. **Feng X., Liu J.** Efficacy and safety of adenosine for supraventricular tachycardia: A meta-analysis utilizing BioMedGPT-LM-7B. *BMC Cardiovascular Disorders*. **25**(1), (2025)

35. **Franzoi D., Bockting C.L., Bennett K.F., Odom A., Lucassen P.J., Pathania A.*, et al.*** Which individual, social, and urban factors in early childhood predict psychopathology in later childhood, adolescence and young adulthood? A systematic review. *SSM Popul Health*. **25**,101575 (2024)

36. **Garcia-Torres D., Vicente Ripoll M.A., Fernandez Peris C., Mira Solves J.J.** Enhancing Clinical Reasoning with Virtual Patients: A Hybrid Systematic Review Combining Human Reviewers and ChatGPT. *Healthcare (Basel)*. **12**(22),11 (2024)

37. **Ghozy S., Amoukhteh M., Hasanzadeh A., Jannatdoust P., Shafie M., Valizadeh P.*, et al.*** Net water uptake as a predictive neuroimaging marker for acute ischemic stroke outcomes: a meta-analysis. *European Radiology*. **34**(8),5308-16 (5308)

38. **Glenn D.L., Choi S.H., Zimmerman R.S.** A systematic review and narrative summary of the therapeutic potential of classic serotonergic psychedelics for smoking cessation and reduction. *Journal of Psychopharmacology*. **39**(9),930-9 (2025)

39. **Goldkuhle M., Dimaki M., Gartlehner G., Monsef I., Dahm P., Glossmann J.P.*, et al.*** Nivolumab for adults with Hodgkin's lymphoma (a rapid review using the software RobotReviewer). *Cochrane Database Syst Rev*. **7**(7),CD012556 (2018)

40. **Grinzinger C., Stuben G., Neu M., Rubeck A., Schiele S., Behrens L.*, et al.*** Effects of concurrent HER2-directed therapy on development of cerebral radionecrosis after stereotactic radiotherapy: a systematic review. *Strahlentherapie und Onkologie*. **201**(9),863-73 (2025)

41. **Halamoda-Kenzaoui B., Rolland E., Piovesan J., Puertas Gallardo A., Bremer-Hoffmann S.** Toxic effects of nanomaterials for health applications: How automation can support a systematic review of the literature? *J Appl Toxicol*. **42**(1),41-51 (2022)

42. **Hollands G.J., Carter P., Anwer S., King S.E., Jebb S.A., Ogilvie D.*, et al.*** Altering the availability or proximity of food, alcohol, and tobacco products to change their selection and consumption. *Cochrane Database Syst Rev*. **9**(9),CD012573 (2019)

43. **Hosseini S., Acar A., Sen M., Meeder K., Singh P., Yin K.*, et al.*** Penetrance of Gastric Adenocarcinoma Susceptibility Genes: A Systematic Review. *Ann Surg Oncol*. **30**(3),1795-807 (2023)

44. **Hsieh P., Apaydin E., Briggs R.G., Al-Amodi D., Aleman A., Dubel K.*, et al.*** Diagnosis and Treatment of Tethered Spinal Cord. *Agency for Healthcare Research and Quality (US)*. **24**(25),10 (2024)

45. **Iaconisi G.N., Ahmed A., Lauria G., Gallo N., Fiermonte G., Cowman M.K.*, et al.*** Targeting mitochondria in Cancer therapy: Machine learning analysis of hyaluronic acid-based drug delivery systems. *International Journal of Biological Macromolecules*. **283**(no pagination), (2024)

46. **Jackson S., Brown J., Norris E., Livingstone-Banks J., Hayes E., Lindson N.** Mindfulness for smoking cessation. *Cochrane Database Syst Rev*. **4**(4),CD013696 (2022)

47. **Jayasundara D., Jayawardane I.A., Denuwara H., Jayasingha T.** Membrane sweeping at term to promote spontaneous labor and reduce the likelihood of formal labor induction for prolonged pregnancy, in South Asia and the world: A meta-analysis. *Int J Gynaecol Obstet*. **166**(2),567-79 (2024)

48. **Jayawardane I.A., Jayasundara D., Weliange S.D.S., Jayasingha T., Madugalle T., Nishshanka N.** Long-term morbidity of peripartum hysterectomy: A systematic review. *International Journal of Gynaecology & Obstetrics*. **170**(3),988-1000 (2025)

49. **Kang F.W., Yuan X.R., Li G.C., Yang Y.F.Z., Zhang X.M., Hou G.Y.** Coronectomy in Lower Third Molar Surgery: A Systematic Review and Meta-Analysis. *Journal of Oral and Maxillofacial Surgery*. **83**(5),601-15 (2025)

50. **Karagiannis T., Andreadis P., Manolopoulos A., Malandris K., Avgerinos I., Karagianni A.*, et al.*** Decision aids for people with Type 2 diabetes mellitus: an effectiveness rapid review and meta-analysis. *Diabet Med*. **36**(5),557-68 (2019)

51. **Kendall N., Hamouda A.M., Cwajna M., Gajjar A., Derhab M., Ghozy S.*, et al.*** Factors associated with ICU stays after endovascular treatments for unruptured intracranial aneurysms: A review study. *Journal of Clinical Neuroscience*. **134**(no pagination), (2025)

52. **Kim H.D., Cruz A.B.** Transformational Leadership and Psychological Well-Being of Service-Oriented Staff: Hybrid Data Synthesis Technique. *Int J Environ Res Public Health*. **19**(13), (2022)

53. **Kumar N., Xu G.D., Lathrop C., Shi J., Kumar A., Winston G.*, et al.*** Impact of social factors on outcomes following pediatric neuro-oncology surgery in the United States: a systematic review and meta-analysis. *Neurosurgical Review*. **48**(1), (2025)

54. **Lam J., Howard B.E., Thayer K., Shah R.R.** Low-calorie sweeteners and health outcomes: A demonstration of rapid evidence mapping (rEM). *Environ Int*. **123**,451-8 (2019)

55. **Landau M., Comeaux M., Mortell T., Boyle R., Imbrescia K., Chaffin A.E.** Characterizing the untapped potential of virtual reality in plastic and reconstructive surgical training: A systematic review on skill transferability. *JPRAS Open*. **41**(pp 295-310), (2024)

56. **Liu Y., Zhao S., Zhang X., Zhang X., Liang T., Ning Z.** The Effects of Imagery Practice on Athletes' Performance: A Multilevel Meta-Analysis with Systematic Review. *Behav Sci (Basel)*. **15**(5),16 (2025)

57. **Lowe D., Ryan R., Schonfeld L., Merner B., Walsh L., Graham-Wisener L.*, et al.*** Effects of consumers and health providers working in partnership on health services planning, delivery and evaluation. *Cochrane Database Syst Rev*. **9**(9),CD013373 (2021)

58. **Marin A., Chiaradia V.C., Dobre M., Brateanu A., Baltatu O.C., Campos L.A.** High-frequency chest wall oscillation devices: An umbrella review and bibliometric analysis. *Computers in Biology and Medicine*. **182**(no pagination), (2024)

59. **McOwiti A.O., Tao W., Tao C.** Identification and classification of principal features for analyzing unwarranted clinical variation. *J Eval Clin Pract*. **30**(2),251-9 (2024)

60. **Meherali S., Nisa S., Aynalem Y.A., Kennedy M., Salami B., Adjorlolo S.*, et al.*** Impact of Climate Change on Adolescents' Health Outcomes: An Evidence Gap Map Review. *Journal of Adolescence*. **97**(3),609-19 (2025)

61. **Melinte M.A., Nistor D.V., de Souza Conde R.A., Hernandez R.G., Wijaya P., Marvin K.*, et al.*** Mini-open versus percutaneous surgical repair for acute Achilles tendon rupture: a systematic review and meta-analysis. *International Orthopaedics*. **49**(1),259-69 (2025)

62. **Merino-Barbancho B., Cipric A., Arroyo P., Rujas M., Gomez del Moral Herranz R.M., Barev T.*, et al.*** Methods and computational techniques for predicting adherence to treatment: A scoping review. *Computers in Biology and Medicine*. **192**(no pagination), (2025)

63. **Miranda L., Paul R., Putz B., Koutsouleris N., Muller-Myhsok B.** Systematic Review of Functional MRI Applications for Psychiatric Disease Subtyping. *Front Psychiatry*. **12**,665536 (2021)

64. **Mushcab H., Al Ramis M., AlRujaib A., Eskandarani R., Sunbul T., AlOtaibi A.*, et al.*** Application of Artificial Intelligence in Cardio-Oncology Imaging for Cancer Therapy-Related Cardiovascular Toxicity: Systematic Review. *JMIR Cancer*. **11**(no pagination), (2025)

65. **Napolitano F., Xu X., Gao X.** Impact of computational approaches in the fight against COVID-19: an AI guided review of 17 000 studies. *Brief Bioinform*. **23**(1), (2022)

66. **Nogueira R., Eguchi M., Kasmirski J., de Lima B.V., Dimatos D.C., Lima D.L.*, et al.*** Machine Learning, Deep Learning, Artificial Intelligence and Aesthetic Plastic Surgery: A Qualitative Systematic Review. *Aesthetic plastic surgery*. **49**(1),389-99 (2025)

67. **Noteboom Y., Montanus A.W.A., van Nassau F., Burchell G., Anema J.R., Huysmans M.A.** Barriers and facilitators of collaboration during the implementation of vocational rehabilitation interventions: a systematic review. *BMC Psychiatry*. **24**(1), (2024)

68. **Olaya-Mira N., Gomez-Hernandez L.M., Viloria-Barragan C., Soto-Cardona I.C.** Methods to assess lower limb prosthetic adaptation: a systematic review. *Journal of NeuroEngineering and Rehabilitation*. **22**(1), (2025)

69. **Petrolini-Mateus A., Araujo G.H.G., Schafauser-Segundo N.S., Leonardi N.T., Castello-Simoes V., Hurst J.R.*, et al.*** Prevalence of chronic respiratory disease using case-finding tools in adults living with noncommunicable disease in low- and middle-income countries: a systematic review. *BMC Pulmonary Medicine*. **25**(1), (2025)

70. **Pillay J., Gaudet L., Wingert A., Bialy L., Mackie A.S., Paterson D.I.*, et al.*** Incidence, risk factors, natural history, and hypothesised mechanisms of myocarditis and pericarditis following covid-19 vaccination: living evidence syntheses and review. *BMJ*. **378**,e069445 (2022)

71. **Pinna F., Manchia M., Paribello P., Carpiniello B.** The Impact of Alexithymia on Treatment Response in Psychiatric Disorders: A Systematic Review. *Front Psychiatry*. **11**,311 (2020)

72. **Rakhshandehroo S., Duits N., Bergman D., Verkes R.J., Kempes M.** Psychopathology in female offenders of terrorism and violent extremism: a systematic review. *Front Psychiatry*. **14**,1123243 (2023)

73. **Riaz I.B., He H., Ryu A.J., Siddiqi R., Naqvi S.A.A., Yao Y.*, et al.*** A Living, Interactive Systematic Review and Network Meta-analysis of First-line Treatment of Metastatic Renal Cell Carcinoma. *Eur Urol*. **80**(6),712-23 (2021)

74. **Robinson C.L., Fonseca A.C.G., Diejomaoh E.M., D'Souza R S., Schatman M.E., Orhurhu V.*, et al.*** Scoping Review: The Role of Psychedelics in the Management of Chronic Pain. *Journal of Pain Research*. **17**(pp 965-973), (2024)

75. **Sarbout I., Gungor A., Ounissi M., Zaher S., Ptito M., Kupers R.*, et al.*** Visual Prostheses in the Era of Artificial Intelligence Technology. *Eye brain*. **17**,95-113 (2025)

76. **Shakeri Hossein Abad Z., Kline A., Sultana M., Noaeen M., Nurmambetova E., Lucini F.*, et al.*** Digital public health surveillance: a systematic scoping review. *NPJ Digit Med*. **4**(1),41 (2021)

77. **Shemilt I., Hollands G.J., Marteau T.M., Nakamura R., Jebb S.A., Kelly M.P.*, et al.*** Economic instruments for population diet and physical activity behaviour change: a systematic scoping review. *PLoS One*. **8**(9),e75070 (2013)

78. **Silva G.F.S., Fagundes T.P., Teixeira B.C., Chiavegatto Filho A.D.P.** Machine Learning for Hypertension Prediction: a Systematic Review. *Curr Hypertens Rep*. **24**(11),523-33 (2022)

79. **Slebe R., Wenker E., Schoonmade L.J., Bouman E.J., Blondin D.P., Campbell D.J.T.*, et al.*** The effect of preprandial versus postprandial physical activity on glycaemia: Meta-analysis of human intervention studies. *Diabetes Res Clin Pract*. **210**,111638 (2024)

80. **Sorrentino M., Belpiede A., Fiorilla C., Mercogliano M., Triassi M., Palladino R.** Logistic and organizational barriers to herpes zoster vaccination in europe: A systematic review. *Vaccine: X*. **20**(no pagination), (2024)

81. **Sorrentino M., Fiorilla C., Mercogliano M., Esposito F., Stilo I., Affinito G.*, et al.*** Technological interventions in European dementia care: a systematic review of acceptance and attitudes among people living with dementia, caregivers, and healthcare workers. *Frontiers in Neurology*. **15**(no pagination), (2024)

82. **Spinelli A., Carrano F.M., Laino M.E., Andreozzi M., Koleth G., Hassan C.*, et al.*** Artificial intelligence in colorectal surgery: an AI-powered systematic review. *Tech Coloproctol*. **27**(8),615-29 (2023)

83. **Steele D.W., Kanaan G., Caputo E.L., Freeman J.B., Brannan E.H., Balk E.M.*, et al.*** Treatment of Obsessive-Compulsive Disorder in Children and Youth: A Meta-Analysis. *Pediatrics*. **155**(3), (2025)

84. **Sun C., Dai H., van der Kleij R.M.J.J., Li R., Wu H., Hallensleben C.*, et al.*** Digital Health Education for Chronic Lung Disease: Scoping Review. *Journal of Medical Internet Research*. **27**(no pagination), (2025)

85. **Susai S., Motwani R., Chandrupatla M.** Tracking Lymphatic Drainage Pathways Through Inner Ear Channels: A Systematic Review. *Cureus*. **16**(8),e66670 (2024)

86. **Talukdar R., Ajayan R., Gupta S., Biswas S., Parveen M., Sadhukhan D.*, et al.*** Chronic Kidney Disease Prevalence in India: A Systematic Review and Meta-Analysis From Community-Based Representative Evidence Between 2011 to 2023. *Nephrology*. **30**(1), (2025)

87. **Teperikidis E., Boulmpou A., Potoupni V., Kundu S., Singh B., Papadopoulos C.** Does the long-term administration of proton pump inhibitors increase the risk of adverse cardiovascular outcomes? A ChatGPT powered umbrella review. *Acta Cardiol*. **78**(9),980-8 (2023)

88. **Tun H.M., Rahman H.A., Naing L., Malik O.A.** Trust in Artificial Intelligence-Based Clinical Decision Support Systems Among Health Care Workers: Systematic Review. *Journal of Medical Internet Research*. **27**(no pagination), (2025)

89. **Valizadeh P., Jannatdoust P., Ghadimi D.J., Bagherieh S., Hassankhani A., Amoukhteh M.*, et al.*** Predicting lymph node metastasis in thyroid cancer: systematic review and meta-analysis on the CT/MRI-based radiomics and deep learning models. *Clinical Imaging*. **119**(no pagination), (2025)

90. **Valizadeh P., Jannatdoust P., Pahlevan-Fallahy M.T., Hassankhani A., Amoukhteh M., Bagherieh S.*, et al.*** Diagnostic accuracy of radiomics and artificial intelligence models in diagnosing lymph node metastasis in head and neck cancers: a systematic review and meta-analysis. *Neuroradiology*. **67**(2),449-67 (2025)

91. **Vallury K.D., Jones M., Oosterbroek C.** Computerized Cognitive Behavior Therapy for Anxiety and Depression in Rural Areas: A Systematic Review. *J Med Internet Res*. **17**(6),e139 (2015)

92. **van den Berg R.L., van der Landen S.M., Keijzer M.J., van Gils A.M., van Dam M., Ziesemer K.A.*, et al.*** Smartphone- and Tablet-Based Tools to Assess Cognition in Individuals With Preclinical Alzheimer Disease and Mild Cognitive Impairment: Scoping Review. *Journal of Medical Internet Research*. **27**(no pagination), (2025)

93. **van Dijk S.H.B., Brusse-Keizer M.G.J., Bucsan C.C., Ploumen E.H., van Beurden W.J.C., van der Palen J.*, et al.*** Lack of Evidence Regarding Markers Identifying Acute Heart Failure in Patients with COPD: An AI-Supported Systematic Review. *Int J Chron Obstruct Pulmon Dis*. **19**,531-41 (2024)

94. **Visser F.C.W., Kloppenburg-Lagendijk M., Hempenius L., Verwey N.A., Perry M., Van Eersel M.E.A.*, et al.*** From suspicion of cognitive decline to dementia diagnosis: A systematic review of healthcare professionals' considerations and attitudes. *Age and Ageing*. **54**(6), (2025)

95. **Vizcarra J.A., Yarlagadda S., Xie K., Ellis C.A., Spindler M., Hammer L.H.** Artificial Intelligence in the Diagnosis and Quantitative Phenotyping of Hyperkinetic Movement Disorders: A Systematic Review. *Journal of Clinical Medicine*. **13**(23), (2024)

96. **Voorn P.B., Oomen R., Buczny J., Bossen D., Visser B., Pijnappels M.** The effect of exercise-induced muscle fatigue on gait parameters among older adults: a systematic review and meta-analysis. *Eur*. **22**(1),4 (2025)

97. **Wen Y., Gu P., Duan Y., Lu Z., Li J., Deng M.*, et al.*** Bayesian Network Meta-Analysis of Randomized Controlled Trials Comparing the Efficacy of Diverse Interventions in Preventing Portal Venous System Thrombosis Following Splenectomy in Patients with Liver Cirrhosis. *Annals of Vascular Surgery*. **121**(pp 299-309), (2025)

98. **Westendorp J., Geerse O.P., van der Lee M.L., Schoones J.W., van Vliet M.H.M., Wit T.*, et al.*** Harmful communication behaviors in cancer care: A systematic review of patients and family caregivers perspectives. *Psychooncology*. **32**(12),1827-38 (2023)

99. **Yamikan H., Ahiskali G.N., Demirel A., Kutukcu E.C.** The effects of exercise-based prehabilitation in patients undergoing coronary artery bypass grafting surgery: A systematic review of randomized controlled trials. *Heart and Lung*. **69**(pp 41-50), (2025)

100. **Yappalparvi A., Balaraman A.K., Padmapriya G., Gaidhane S., Kaur I., Lal M.*, et al.*** Safety and efficacy of ensifentrine in COPD: A systemic review and meta-analysis. *Respiratory Medicine*. **236**(no pagination), (2025)

101. **Yazicioglu I., Bagcivan G., Kolic A.** The Cost-Effectiveness of Nursing Interventions in Cancer Care: Systematic Review. *Seminars in Oncology Nursing*. **41**(4), (2025)

102. **Zamantakis A., Merle J.L., Queiroz A.A., Zapata J.P., Deskins J., Pachicano A.M.*, et al.*** Innovation and implementation determinants of HIV testing and linkage-to-care in the U.S.: a systematic review. *Implement*. **5**(1),111 (2024)

103. **Zhu E.M., Buljac-Samardzic M., Ahaus K., Sevdalis N., Huijsman R.** Implementation and dissemination of home- and community-based interventions for informal caregivers of people living with dementia: a systematic scoping review. *Implement Sci*. **18**(1),60 (2023)

104. **Crossingham I., Turner S., Ramakrishnan S., Fries A., Gowell M., Yasmin F.*, et al.*** Combination fixed-dose beta agonist and steroid inhaler as required for adults or children with mild asthma. *Cochrane Database Syst Rev*. **5**(5),CD013518 (2021)

105. **Eun M.Y., Jeon E.T., Seo K.D., Lee D., Jung J.M.** Reperfusion Therapy in Acute Ischemic Stroke with Active Cancer: A Meta-Analysis Aided by Machine Learning. *J Stroke Cerebrovasc Dis*. **30**(6),105742 (2021)

106. **Foulquier N., Redou P., Le Gal C., Rouviere B., Pers J.O., Saraux A.** Pathogenesis-based treatments in primary Sjogren's syndrome using artificial intelligence and advanced machine learning techniques: a systematic literature review. *Hum Vaccin Immunother*. **14**(11),2553-8 (2018)

107. **Gaskins N.J., Bray E., Hill J.E., Doherty P.J., Harrison A., Connell L.A.** Factors influencing implementation of aerobic exercise after stroke: a systematic review. *Disabil Rehabil*. **43**(17),2382-96 (2021)

108. **Giummarra M.J., Lau G., Grant G., Gabbe B.J.** A systematic review of the association between fault or blame-related attributions and procedures after transport injury and health and work-related outcomes. *Accid Anal Prev*. **135**,105333 (2020)

109. **Rogers C.R., Matthews P., Xu L., Boucher K., Riley C., Huntington M.*, et al.*** Interventions for increasing colorectal cancer screening uptake among African-American men: A systematic review and meta-analysis. *PLoS One*. **15**(9),e0238354 (2020)

110. **Viner R., Russell S., Saulle R., Croker H., Stansfield C., Packer J.*, et al.*** School Closures During Social Lockdown and Mental Health, Health Behaviors, and Well-being Among Children and Adolescents During the First COVID-19 Wave: A Systematic Review. *JAMA Pediatr*. **176**(4),400-9 (2022)

111. **Xiong Z., Liu T., Tse G., Gong M., Gladding P.A., Smaill B.H.*, et al.*** A Machine Learning Aided Systematic Review and Meta-Analysis of the Relative Risk of Atrial Fibrillation in Patients With Diabetes Mellitus. *Front Physiol*. **9**,835 (2018)

112. **Yamamoto R., Ito T., Nagasawa Y., Matsui K., Egawa M., Nanami M.*, et al.*** Efficacy of aerobic exercise on the cardiometabolic and renal outcomes in patients with chronic kidney disease: a systematic review of randomized controlled trials. *J Nephrol*. **34**(1),155-64 (2021)

113. **Agency C.s.D.** Position statement on the use of artificial intelligence in the generation and reporting of evidence [Internet]. [cited 2025 November 14]. Available from: https://www.cda-amc.ca.,

114. **IQWiG**. General Methods—Version 7.0. Available at https://www.iqwig.de/methoden/general-methods_version-7-0.pdf. (2023)

115. **NICE**. Use of AI in evidence generation: NICE position statement. Available from: https://www.nice.org.uk/about/what-we-do/our-research-work/use-of-ai-in-evidence-generation--nice-position-statement. (Accessed February 2024).

116. **EUnetHTA JA3WP6B2-2 Authoring Team.** Process of information retrieval for systematic reviews and health technology assessments on clinical effectiveness (version 2.0) (Methodological Guidelines). Available at https://www.eunethta.eu/wp-content/uploads/2020/01/EUnetHTA_Guideline_Information_Retrieval_v2-0.pdf

(2019)

117. Cochrane handbook for systematic reviews of interventions version 6.5 (updated August 2024). Higgins H, Thomas J, Chandler J, Cumpston M, Li T, Page M, et al., editors. Cochrane training: Cochrane; 2024.

118. Aromataris E., Lockwood C., Porritt K., Pilla B., Jordan Z., Editors. JBI Manual for Evidence Synthesis.; 2024.

## Supplementary Table 1. Embase and Ovid MEDLINE(R) searches

### Original search (ran on June 25, 2024)

| **#** | **Searches** | **Hits** | |
| --- | --- | --- | --- |
|  |  | **Embase** | **Ovid MEDLINE(R)** |
| **1** | artificial intelligence.ti,ab. or exp artificial intelligence/ or ML.ti,ab. or exp ML/ or ML algorithm.ti,ab. or deep learning.ti,ab. or automat$.ti,ab. or semi-automat$.ti,ab. or text mining.ab,ti. or text-mining,ab.ti. or exp NLP/ or NLP.ti,ab. or large language model.ti,ab. or generative AI.ti,ab. or generative artificial intelligence.ti,ab. or AI agent$.ti,ab. or agent AI.ti,ab. | 916,231 | 567,609 |
| **2** | (Abstrackr or ASReview or Bard or Bioreader or Carrot2 or ChatGPT or Claude or Colandr or Covidence or CrowdCARE or Data Abstraction Assistant or DistillerSR or Dragon or DAISY or EPPI-Reviewer or GAPScreener or Gemini or JBI-SUMARI or Leximancer or Lingo3G or MAVIS or meta-analysis via Shiny or Medical Text Indexer or Medline Ranker or National Centre for Text Mining or PubReMiner or OpenAI or RCT classifier or RCT tagger or Rayyan or ReVis or revtools or RobotAnalyst or RobotReviewer or robvis or SWIFT-Active Screener or SWIFT-Review or SensPrecOptimizer or sysrev or TerMine or Textpresso or Trial2Rev or Voyant or WordStat).ab,ti. | 17,472 | 13,573 |
| **3** | 1 or 2 | 930,303 | 578,712 |
| **4** | systematic review.mp. or exp systematic review/ or systematic literature review.mp. or meta-analysis.mp. or exp meta analysis/ | 776,043 | 494,207 |
| **5** | (animal cell or animal experiment or animal model or cell culture or tissue culture or in vitro or nonhuman or biological model).ab,ti. | 2,076,197 | 1,679,255 |
| **6** | 3 and 4 | 17,450 | 8527 |
| **7** | 6 not 5 | 17,196 | 8432 |
| **8** | (case stud$ or case report$ or case series or guideline$ or practice guideline$ or validation stud$).ti. | 690,449 | 554,960 |
| **9** | 7 not 8 | 16,951 | 8333 |
| **10** | limit 9 to (human and english language and "review") | 6324 | 2346 |

### Updated search (ran on September 9, 2025)

| **#** | **Searches** | **Hits** | |
| --- | --- | --- | --- |
|  |  | **Embase** | **Ovid MEDLINE(R)** |
| **1** | artificial intelligence.ti,ab. or exp artificial intelligence/ or ML.ti,ab. or exp ML/ or ML algorithm.ti,ab. or deep learning.ti,ab. or automat$.ti,ab. or semi-automat$.ti,ab. or text mining.ab,ti. or text-mining,ab.ti. or exp NLP/ or NLP.ti,ab. or large language model.ti,ab. or generative AI.ti,ab. or generative artificial intelligence.ti,ab. or AI agent$.ti,ab. or agent AI.ti,ab. | 2,175,412 | 1,577,312 |
| **2** | (Abstrackr or ASReview or Bard or Bioreader or Carrot2 or ChatGPT or Claude or Colandr or Covidence or CrowdCARE or Data Abstraction Assistant or DistillerSR or Dragon or DAISY or EPPI-Reviewer or GAPScreener or Gemini or JBI-SUMARI or Leximancer or Lingo3G or MAVIS or meta-analysis via Shiny or Medical Text Indexer or Medline Ranker or National Centre for Text Mining or PubReMiner or OpenAI or RCT classifier or RCT tagger or Rayyan or ReVis or revtools or RobotAnalyst or RobotReviewer or robvis or SWIFT-Active Screener or SWIFT-Review or SensPrecOptimizer or sysrev or TerMine or Textpresso or Trial2Rev or Voyant or WordStat).ab,ti. | 24,399 | 19,232 |
| **3** | 1 or 2 | 2,190,708 | 1,590,596 |
| **4** | systematic review.mp. or exp systematic review/ or systematic literature review.mp. or meta-analysis.mp. or exp meta analysis/ | 880,531 | 561,325 |
| **5** | (animal cell or animal experiment or animal model or cell culture or tissue culture or in vitro or nonhuman or biological model).ab,ti. | 2,202,989 | 1,774,250 |
| **6** | 3 and 4 | 30,327 | 18,800 |
| **7** | 6 not 5 | 29,872 | 18,570 |
| **8** | (case stud$ or case report$ or case series or guideline$ or practice guideline$ or validation stud$).ti. | 754,932 | 613,187 |
| **9** | 7 not 8 | 29,195 | 18,310 |
| **10** | limit 9 to (human and english language and "review") | 9959 | 3401 |
| **11** | Limit 10 to yr=”2024 -Current” | 3391 | 1574 |

## Supplementary Table 2. Cochrane Reviews searches

### Original search (ran on June 25, 2024)

| **#** | **Searches** | **Hits** |
| --- | --- | --- |
| **1** | MeSH descriptor: [Artificial Intelligence] explode all trees | 3198 |
| **2** | ("systematic review" OR "scoping review" OR "integrative review" OR "umbrella review" OR "rapid review" or "meta analysis"):ti,ab,kw | 27,471 |
| **3** | (Abstrackr or ASReview or Bard or Bioreader or Carrot2 or ChatGPT or Claude or Colandr or Covidence or CrowdCARE or "Data Abstraction Assistant" or DistillerSR or Dragon or DAISY or EPPI-Reviewer or GAPScreener or Gemini or JBI-SUMARI or Leximancer or Lingo3G or MAVIS or "meta-analysis via Shiny" or "Medical Text Indexer" or "Medline Ranker" or "National Centre for Text Mining" or OpenAI or PubReMiner or "RCT classifier" or "RCT tagger" or Rayyan or ReVis or revtools or RobotAnalyst or RobotReviewer or robvis or "SWIFT-Active Screener" or SWIFT-Review or SensPrecOptimizer or sysrev or TerMine or Textpresso or Trial2Rev or Voyant or WordStat):ab | 242 |
| **4** | #4 "ML" NEAR/20 review OR "artificial intelligence" NEAR/20 review OR automat* NEAR/10 review OR semi-automat* NEAR/10 review OR "text mining" NEAR/20 review OR "NLP" NEAR/20 review OR "large language model" NEAR/20 review OR "generative AI" NEAR/20 review OR "generative artificial intelligence" NEAR/20 review OR "AI agent" NEAR/20 review OR "agent AI" NEAR/20 review | 92 |
| **5** | 1 OR 3 | 250 |
| **6** | 2 AND 5 | 128 |
| **7** | 4 OR 6 | 226 |

### Updated search (ran on September 9, 2025)

| **#** | **Searches** | **Hits** |
| --- | --- | --- |
| **1** | MeSH descriptor: [Artificial Intelligence] explode all trees | 3783 |
| **2** | ("systematic review" OR "scoping review" OR "integrative review" OR "umbrella review" OR "rapid review" or "meta analysis"):ti,ab,kw | 28,966 |
| **3** | (Abstrackr or ASReview or Bard or Bioreader or Carrot2 or ChatGPT or Claude or Colandr or Covidence or CrowdCARE or "Data Abstraction Assistant" or DistillerSR or Dragon or DAISY or EPPI-Reviewer or GAPScreener or Gemini or JBI-SUMARI or Leximancer or Lingo3G or MAVIS or "meta-analysis via Shiny" or "Medical Text Indexer" or "Medline Ranker" or "National Centre for Text Mining" or OpenAI or PubReMiner or "RCT classifier" or "RCT tagger" or Rayyan or ReVis or revtools or RobotAnalyst or RobotReviewer or robvis or "SWIFT-Active Screener" or SWIFT-Review or SensPrecOptimizer or sysrev or TerMine or Textpresso or Trial2Rev or Voyant or WordStat):ab | 1045 |
| **4** | #4 "ML" NEAR/20 review OR "artificial intelligence" NEAR/20 review OR automat* NEAR/10 review OR semi-automat* NEAR/10 review OR "text mining" NEAR/20 review OR "NLP" NEAR/20 review OR "large language model" NEAR/20 review OR "generative AI" NEAR/20 review OR "generative artificial intelligence" NEAR/20 review OR "AI agent" NEAR/20 review OR "agent AI" NEAR/20 review | 2346 |
| **5** | 1 OR 3 | 4779 |
| **6** | 2 AND 5 | 89 |
| **7** | 4 OR 6 (with Cochrane Library publication date from May 2024 to Sep 2025) | 365 |

## Supplementary Table 3. Risk of bias assessment details

|  | **Study eligibility criteria** | | | | | | **Identification and selection of studies** | | | | | | **Data collection and study appraisal** | | | | | |
| --- | --- | --- | --- | --- | --- | --- | --- | --- | --- | --- | --- | --- | --- | --- | --- | --- | --- | --- |
|  | 1.1 | 1.2 | 1.3 | 1.4 | 1.5 |  | 2.1 | 2.2 | 2.3 | 2.4 | 2.5 |  | 3.1 | 3.2 | 3.3 | 3.4 | 3.5 |  |
| **Aali et al. 2020 (1)** | PN | PY | PY | PY | PY | *High* | PY | Y | PY | N | Y | *High* | Y | Y | PY | PY | PN | *High* |
| **Agarwal et al. 2021 (2)** | Y | Y | Y | PY | Y | *Low* | Y | Y | Y | PY | PY | *Low* | Y | Y | Y | Y | Y | *Low* |
| **Al-Obeidat et al., 2024 (3)** | Y | Y | Y | Y | PY | *Low* | Y | N | Y | PY | Y | *High* | Y | Y | Y | Y | Y | *Low* |
| **Al-Sammarraie et al., 2025 (4)** | Y | Y | Y | Y | PY | *Low* | Y | Y | Y | Y | Y | *Low* | PY | Y | Y | N | N | *High* |
| **Albuquerque *et al*., 2025 (5)** | Y | Y | Y | Y | PY | *Low* | Y | N | Y | PY | PY | *High* | PY | Y | Y | N | N | *High* |
| **Alfredo Ardisson Cirino Campos et al., 2025 (6)** | Y | Y | Y | Y | PY | *Low* | Y | Y | Y | PY | Y | *Low* | Y | Y | Y | N | PY | *High* |
| **Anggreni et al., 2025 (7)** | Y | Y | Y | Y | PY | *Low* | Y | N | PY | PY | Y | *High* | Y | Y | Y | Y | Y | *Low* |
| **Aucoin et al. 2020 (8)** | PN | PY | PY | Y | PN | *High* | Y | Y | Y | Y | PY | *Low* | NI | PN | PY | NI | NI | *High* |
| **Aujla et al., 2024 (9)** | Y | Y | Y | Y | PY | *Low* | Y | N | Y | Y | Y | *High* | Y | Y | Y | Y | PY | *Low* |
| **Bagg et al., 2024 (10)** | Y | PY | PY | PY | PY | *Low* | Y | N | Y | Y | PN | *High* | N | Y | PY | N | N | *High* |
| **Balk et al. 2023a (11)** | Y | PY | Y | PY | Y | *Low* | Y | Y | Y | PY | Y | *Low* | PY | Y | PY | Y | Y | *Low* |
| **Balk et al. 2023b (12)** | Y | PY | Y | PY | Y | *Low* | Y | Y | Y | PY | Y | *Low* | PY | Y | PY | Y | Y | *Low* |
| **Baron et al. 2013 (13)** | N | PY | PN | PY | PN | *High* | Y | N | PY | PN | PY | *High* | NI | N | PY | NI | NI | *High* |
| **Bell-Aldeghi et al. 2023 (14)** | Y | Y | Y | PY | PN | *High* | Y | N | PY | Y | Y | *High* | PY | PY | PY | NI | NI | *Unclear* |
| **Bilal et al. 2018 (15)** | PN | PY | N | PY | PY | *High* | Y | Y | NI | PY | Y | *Unclear* | Y | Y | PY | Y | NI | *Unclear* |
| **Briand et al. 2025 (16)** | Y | Y | Y | PY | PY | *Low* | Y | Y | Y | PY | Y | *Low* | Y | Y | Y | Y | Y | *Low* |
| **Brons et al., 2024 (17)** | Y | Y | Y | Y | Y | *Low* | Y | Y | Y | Y | Y | *Low* | Y | Y | Y | N | PY | *High* |
| **Brozek et al. 2024 (18)** | Y | PY | PY | PY | Y | *Low* | Y | Y | PY | Y | Y | *Low* | Y | PY | PY | PY | PY | *Low* |
| **Buchlak et al. 2022 (19)** | PN | PY | PY | PN | PN | *High* | Y | Y | NI | NI | PY | *Unclear* | NI | PY | PY | Y | NI | *Unclear* |
| **Buchlak et al. 2021 (20)** | PY | PY | PY | PY | PN | *High* | Y | Y | PY | NI | PY | *Unclear* | NI | PY | PY | Y | NI | *Unclear* |
| **Buchlak et al. 2020 (21)** | PN | PY | PY | PY | PN | *High* | Y | Y | PY | NI | PY | *Unclear* | NI | PY | PY | Y | NI | *Unclear* |
| **Burger et al., 2025 (22)** | Y | Y | Y | Y | Y | *Low* | Y | Y | Y | Y | Y | *Low* | Y | Y | Y | Y | PY | *Low* |
| **Canova et al., 2024 (23)** | Y | Y | Y | Y | Y | *Low* | Y | Y | Y | Y | PY | *Low* | Y | Y | Y | Y | Y | *Low* |
| **Carlson et al. 2023 (24)** | Y | PY | Y | PY | PY | *Low* | PY | Y | PY | Y | Y | *Low* | Y | PY | PY | N | N | *High* |
| **Chamseddine et al. 2022 (25)** | PN | PY | PN | PY | PN | *High* | N | N | PY | PY | Y | *High* | NI | PY | PY | PY | NI | *Unclear* |
| **da Silva Mulder et al., 2024 (26)** | Y | Y | Y | Y | Y | *Low* | Y | Y | Y | Y | Y | *Low* | Y | Y | Y | Y | Y | *Low* |
| **da Silva et al., 2025 (27)** | Y | Y | Y | Y | PY | *Low* | Y | N | Y | Y | Y | *High* | Y | Y | Y | Y | Y | *Low* |
| **Das et al., 2024 (28)** | Y | Y | Y | Y | Y | *Low* | Y | Y | Y | Y | Y | *Low* | Y | Y | Y | Y | PY | *Low* |
| **de Gans et al. 2024 (29)** | Y | PY | Y | PY | PY | *Low* | Y | Y | PY | Y | PY | *Low* | Y | PY | PY | Y | Y | *Low* |
| **De Zwart & Ruis, 2024 (30)** | PY | PY | PY | PY | PN | *High* | Y | Y | PY | PN | NI | *High* | NI | PY | PY | NI | NI | *Unclear* |
| **Demirtas Yilmaz., 2025 (31)** | Y | Y | Y | Y | PY | *Low* | Y | N | PY | PY | N | *High* | N | Y | PY | N | N | *High* |
| **Ducreux et al., 2025 (32)** | Y | Y | Y | Y | Y | *Low* | Y | N | Y | Y | Y | *High* | Y | Y | Y | Y | Y | *Low* |
| **Feng Q et al., 2025 (33)** | Y | Y | Y | Y | Y | *Low* | Y | PY | Y | Y | Y | *Low* | Y | Y | Y | N | N | *High* |
| **Feng X et al., 2025 (34)** | Y | Y | Y | Y | Y | *Low* | Y | PY | Y | Y | Y | *Low* | Y | Y | Y | Y | Y | *Low* |
| **Franzoi et al. 2024 (35)** | Y | PY | PY | PY | PY | *Low* | Y | Y | PY | PN | Y | *High* | Y | PY | PY | Y | Y | *Low* |
| **García-Torres et al., 2024 (36)** | Y | Y | Y | Y | Y | *Low* | Y | Y | Y | Y | Y | *Low* | Y | Y | Y | Y | Y | *Low* |
| **Ghozy et al., 2024 (37)** | Y | Y | Y | Y | Y | *Low* | Y | Y | Y | Y | Y | *Low* | Y | Y | Y | Y | Y | *Low* |
| **Glenn et al., 2025 (38)** | Y | Y | Y | Y | Y | *Low* | Y | Y | Y | Y | Y | *Low* | Y | Y | Y | Y | Y | *Low* |
| **Goldkuhle et al. 2018 (39)** | Y | PY | PY | PY | PY | *Low* | Y | Y | PY | Y | Y | *Low* | Y | Y | PY | Y | Y | *Low* |
| **Grinzinger et al., 2025 (40)** | Y | Y | Y | Y | Y | *Low* | Y | Y | Y | Y | N | *High* | N | Y | Y | N | N | *High* |
| **Halamoda‐Kenzaoui et al. 2022 (41)** | PN | NI | PN | NI | PN | *High* | N | Y | PY | PY | NI | *High* | PN | PN | PY | NI | NI | *High* |
| **Hollands et al. 2019 (42)** | Y | PY | Y | PY | Y | *Low* | Y | Y | PY | Y | Y | *Low* | Y | Y | PY | Y | Y | *Low* |
| **Hosseini et al. 2023 (43)** | PN | PY | PY | PY | PN | *High* | N | Y | PY | PY | PY | *High* | NI | PY | PY | NI | NI | *Unclear* |
| **Hsieh et al., 2024 (44)** | Y | Y | Y | Y | Y | *Low* | Y | Y | Y | Y | Y | *Low* | Y | Y | Y | Y | Y | *Low* |
| **Iaconisi et al., 2024 (45)** | Y | Y | Y | Y | Y | *Low* | Y | PY | Y | PY | PY | *Low* | PY | Y | Y | N | N | *High* |
| **Jackson et al. 2022 (46)** | Y | PY | Y | PY | Y | *Low* | Y | Y | PY | Y | Y | *Low* | Y | Y | PY | Y | Y | *Low* |
| **Jayasundara et al. 2024 (47)** | PN | PY | Y | PY | PN | *High* | PY | N | PY | PN | Y | *High* | PY | PY | PY | Y | NI | *Unclear* |
| **Jayawardane et al., 2025 (48)** | Y | Y | PY | Y | PY | *Low* | Y | N | Y | PY | Y | *High* | Y | Y | Y | Y | Y | *Low* |
| **Kang et al., 2025 (49)** | Y | Y | Y | Y | PY | *Low* | Y | Y | Y | PY | Y | *Low* | Y | Y | Y | Y | Y | *Low* |
| **Karagiannis et al. 2019 (50)** | Y | PY | Y | PY | PY | *Low* | PN | Y | PY | PY | PY | *High* | PY | PY | PY | PY | PY | *Low* |
| **Kendall et al., 2025 (51)** | Y | Y | Y | Y | PY | *Low* | Y | PY | Y | PY | Y | *Low* | Y | Y | Y | Y | Y | *Low* |
| **Kim & Cruz, 2022 (52)** | PN | PY | PY | PY | PN | *High* | PY | Y | NI | PN | PY | *High* | NI | PY | PY | NI | NI | *Unclear* |
| **Kumar et al., 2025 (53)** | Y | Y | Y | Y | Y | *Low* | Y | N | Y | Y | Y | *High* | Y | Y | Y | Y | Y | *Low* |
| **Lam et al. 2019 (54)** | PY | PY | PY | PY | PY | *Low* | N | N | PY | PY | Y | *High* | PY | PY | PN | N | N | *High* |
| **Landau et al., 2024 (55)** | Y | Y | Y | Y | PY | *Low* | Y | N | Y | PY | Y | *High* | Y | Y | Y | N | N | *High* |
| **Liu Y et al., 2025 (56)** | Y | Y | Y | Y | Y | *Low* | Y | Y | Y | Y | Y | *Low* | Y | Y | Y | Y | Y | *Low* |
| **Lowe et al. 2021 (57)** | Y | PY | Y | PY | PY | *Low* | Y | Y | PY | PN | Y | *High* | Y | PY | PY | Y | Y | *Low* |
| **Marin et al., 2024 (58)** | Y | Y | Y | Y | PY | *Low* | Y | Y | Y | Y | Y | *Low* | Y | Y | Y | Y | Y | *Low* |
| **McOwiti et al. 2024 (59)** | PN | PY | PY | PY | PN | *High* | Y | N | PY | PY | PY | *High* | PY | PN | PY | NI | NI | *High* |
| **Meherali et al., 2025 (60)** | Y | Y | Y | Y | PY | *Low* | Y | Y | Y | Y | Y | *Low* | Y | Y | PY | Y | Y | *Low* |
| **Melinte et al., 2025 (61)** | Y | Y | Y | Y | Y | *Low* | Y | Y | Y | Y | Y | *Low* | Y | Y | Y | Y | Y | *Low* |
| **Merino-Barbancho et al., 2025 (62)** | Y | Y | Y | PY | PY | *Low* | Y | NI | Y | PY | PN | *High* | PY | Y | PY | N | NI | *High* |
| **Miranda et al. 2021 (63)** | PN | PY | PY | PY | Y | *High* | N | N | PY | Y | NI | *High* | NI | PY | PY | NI | NI | *Unclear* |
| **Mushcab et al., 2025 (64)** | Y | Y | Y | Y | Y | *Low* | Y | Y | Y | Y | Y | *Low* | Y | Y | Y | Y | Y | *Low* |
| **Napolitano et al., 2022 (65)** | PN | NI | PN | NI | NI | *High* | PN | Y | PY | PY | NI | *High* | NI | PY | PY | NI | NI | *Unclear* |
| **Nogueira et al., 2025 (66)** | Y | Y | Y | Y | Y | *Low* | Y | Y | Y | Y | Y | *Low* | Y | Y | Y | Y | Y | *Low* |
| **Noteboom et al., 2024 (67)** | Y | Y | Y | Y | Y | *Low* | Y | Y | Y | Y | Y | *Low* | Y | Y | Y | Y | Y | *Low* |
| **Olaya-Mira et al., 2025 (68)** | Y | Y | Y | Y | Y | *Low* | Y | N | Y | Y | Y | *High* | Y | Y | Y | N | N | *High* |
| **Petrolini-Mateus et al., 2025 (69)** | Y | Y | Y | Y | Y | *Low* | Y | N | Y | Y | Y | *High* | Y | Y | Y | Y | Y | *Low* |
| **Pillay et al. 2022 (70)** | PY | PY | Y | PY | PN | *High* | Y | Y | PY | Y | Y | *Low* | Y | PY | PY | Y | Y | *Low* |
| **Pinna et al. 2020 (71)** | PY | PY | PY | PY | PN | *High* | PY | Y | PY | Y | Y | *Low* | NI | PY | PY | Y | NI | *Unclear* |
| **Rakhshandehroo et al. 2023 (72)** | Y | PY | PY | PY | PY | *Low* | Y | Y | PY | PY | Y | *Low* | Y | PY | PY | Y | NI | *Unclear* |
| **Riaz et al. 2021 (73)** | PN | PY | PY | PY | Y | *High* | Y | PY | PY | Y | NI | *Unclear* | NI | PY | PY | Y | NI | *Unclear* |
| **Robinson et al., 2024 (74)** | Y | Y | Y | Y | Y | *Low* | Y | Y | Y | Y | PY | *High* | PY | Y | Y | N | PY | *High* |
| **Sarbout et al., 2025 (75)** | Y | Y | Y | Y | Y | *Low* | Y | N | Y | N | Y | *High* | Y | Y | Y | N | N | *High* |
| **Shakeri Hossein Abad et al. 2021 (76)** | PN | PY | PY | PY | PN | *High* | PY | Y | PY | PY | PN | *High* | Y | PY | PY | NI | NI | *Unclear* |
| **Shemilt et al. 2013 (77)** | PY | PY | PY | PY | PY | *Low* | Y | Y | PY | Y | PN | *High* | N | PY | PY | N | N | *High* |
| **Silva et al. 2022 (78)** | PN | PY | PY | PY | PY | *High* | Y | PN | PY | PN | Y | *High* | NI | PY | PY | NI | NI | *Unclear* |
| **Slebe et al. 2024 (79)** | Y | PY | Y | PY | PY | *Low* | Y | Y | PY | PY | PY | *Low* | PY | PY | PY | Y | Y | *Low* |
| **Sorrentino et al., 2024a (80)** | Y | Y | Y | Y | PY | *Low* | Y | Y | Y | Y | Y | *Low* | Y | Y | Y | Y | Y | *Low* |
| **Sorrentino et al., 2024b (81)** | Y | Y | Y | Y | PY | *Low* | Y | Y | Y | Y | Y | *Low* | Y | Y | Y | Y | Y | *Low* |
| **Spinelli et al. 2023 (82)** | PN | PY | PY | PY | PN | *High* | Y | Y | NI | NI | Y | *Unclear* | Y | PY | PY | Y | Y | *Low* |
| **Steele et al., 2025 (83)** | Y | Y | Y | Y | NI | *Unclear* | Y | Y | Y | NI | Y | *Unclear* | Y | Y | Y | Y | Y | *Low* |
| **Sun et al., 2025 (84)** | Y | Y | Y | Y | Y | *Low* | Y | Y | Y | Y | PY | *High* | Y | Y | Y | Y | PY | *Low* |
| **Susai et al., 2024 (85)** | Y | Y | Y | Y | Y | *Low* | Y | N | PY | Y | Y | *High* | Y | Y | Y | N | N | *High* |
| **Talukdar et al., 2024 (86)** | Y | Y | Y | Y | PY | *Low* | Y | Y | Y | PY | Y | *Low* | Y | Y | Y | Y | Y | *Low* |
| **Teperikidis et al. 2023 (87)** | PN | PY | PY | PY | PN | *High* | N | N | PY | Y | Y | *High* | N | PY | PY | Y | Y | *High* |
| **Tun et al., 2025 (88)** | Y | Y | Y | Y | PY | *Low* | PY | PY | Y | PN | Y | *High* | PY | Y | Y | Y | Y | *Low* |
| **Valizadeh et al., 2025a (89)** | Y | Y | Y | Y | Y | *Low* | Y | Y | Y | Y | Y | *Low* | Y | Y | Y | Y | PY | *Low* |
| **Valizadeh et al., 2025b (90)** | Y | Y | Y | Y | Y | *Low* | Y | Y | Y | Y | Y | *Low* | Y | Y | Y | Y | PY | *Low* |
| **Vallury et al. 2015 (91)** | PN | PY | PY | PY | PN | *High* | Y | Y | PY | Y | PY | *Low* | NI | PY | PY | Y | NI | *Unclear* |
| **van den Berg et al., 2025 (92)** | Y | Y | Y | Y | Y | *Low* | Y | Y | Y | Y | Y | *Low* | Y | Y | Y | PY | Y | *Low* |
| **Van Dijk et al. 2024 (93)** | Y | PY | PY | PY | Y | *Low* | Y | Y | PY | Y | PY | *Low* | PY | PY | PY | Y | Y | *Low* |
| **Visser et al., 2025 (94)** | Y | Y | Y | Y | Y | *Low* | Y | Y | Y | Y | Y | *Low* | Y | Y | Y | Y | Y | *Low* |
| **Vizcarra et al., 2024 (95)** | Y | Y | Y | PY | PY | *Low* | Y | Y | Y | PY | Y | *Low* | Y | Y | PY | Y | Y | *Low* |
| **Voorn et al., 2025 (96)** | Y | Y | Y | Y | PY | *Low* | Y | Y | Y | PY | Y | *Low* | Y | Y | Y | Y | Y | *Low* |
| **Wen et al., 2025 (97)** | Y | Y | Y | Y | PY | *Low* | Y | Y | Y | Y | Y | *Low* | Y | Y | Y | Y | Y | *Low* |
| **Westendorp et al. 2023 (98)** | Y | PY | PY | PY | PN | *High* | Y | Y | PY | Y | PY | *Low* | Y | PY | PY | Y | Y | *Low* |
| **Yamikan et al., 2025 (99)** | Y | Y | Y | Y | PY | *Low* | Y | Y | Y | Y | Y | *Low* | Y | Y | Y | Y | Y | *Low* |
| **Yappalparvi et al. 2025 (100)** | Y | Y | Y | Y | Y | *Low* | Y | N | Y | Y | PY | *High* | Y | Y | Y | N | N | *High* |
| **Yazicioglu et al., 2025 (101)** | Y | Y | Y | Y | PY | *Low* | Y | Y | Y | PY | Y | *Low* | Y | Y | Y | Y | Y | *Low* |
| **Zamantakis et al., 2025 (102)** | Y | Y | Y | Y | PY | *Low* | Y | Y | Y | PY | Y | *Low* | Y | Y | Y | N | PY | *High* |
| **Zhu et al. 2023 (103)** | Y | PY | PY | Y | Y | *Low* | Y | Y | PY | PN | PY | *High* | Y | PY | PY | Y | Y | *Low* |
| **Crossingham et al 2021 (104)** | Y | PY | PY | PY | Y | *Low* | Y | Y | PY | Y | Y | *Low* | Y | PY | PY | Y | Y | *Low* |
| **Eun et al 2021 (105)** | PN | PY | PY | PY | PY | *High* | Y | Y | NI | PY | Y | *Unclear* | NI | PY | PY | Y | Y | *Unclear* |
| **Foulquier et al 2018 (106)** | PN | PY | PN | PN | PN | *High* | N | N | PY | Y | PN | *High* | NI | PY | PY | NI | NI | *Unclear* |
| **Gaskins et al 2021 (107)** | Y | PY | PY | PY | PY | *Low* | Y | Y | PY | PN | N | *High* | PY | PY | PY | N | N | *High* |
| **Giummarra et al 2020 (108)** | Y | PY | PY | PY | PN | *High* | Y | N | PY | PN | Y | *High* | Y | PY | PY | Y | Y | *Low* |
| **Rogers et al 2020 (109)** | Y | PY | PY | PY | PY | *Low* | Y | PY | PY | Y | Y | *Low* | Y | PY | PY | Y | Y | *Low* |
| **Viner et al 2022 (110)** | Y | PY | PY | PY | PN | *High* | Y | Y | PY | PY | Y | *Low* | Y | PY | PY | Y | Y | *Low* |
| **Xiong et al 2018 (111)** | PN | PY | PY | PY | PY | *High* | PN | N | PY | PY | Y | *High* | NI | PY | PY | Y | NI | *Unclear* |
| **Yamamoto et al 2021 (112)** | PN | PY | PY | PY | PN | *High* | N | N | PY | Y | Y | *High* | Y | PY | PY | Y | Y | *Low* |

|  | **Synthesis and findings** | | | | | | | **Risk of bias in the review** | | | |  |
| --- | --- | --- | --- | --- | --- | --- | --- | --- | --- | --- | --- | --- |
|  | 4.1 | 4.2 | 4.3 | 4.4 | 4.5 | 4.6 |  | A | B | C |  |  |
| **Aali et al. 2020 (1)** | Y | PY | Y | Y | PY | PY | *Low* | PN | PY | Y | **High** | A number of concerns identified in domains 1-3 |
| **Agarwal et al. 2021 (2)** | Y | Y | PY | Y | PY | Y | *Low* | Y | Y | Y | **Low** | No concerns identified |
| **Al-Obeidat et al., 2024 (3)** | Y | Y | Y | Y | Y | Y | *Low* | Y | Y | Y | **Low** | Robust study despite lack of supplementary searches |
| **Al-Sammarraie et al., 2025 (4)** | Y | Y | Y | PY | N | PY | *High* | Y | Y | Y | **High** | A number of concerns identified across domains, notably lack of formal RoB assessment |
| **Albuquerque *et al*., 2025 (5)** | Y | Y | Y | PY | N | PY | *High* | Y | Y | Y | **High** | A number of concerns identified across domains - notably, over-reliance on automation. |
| **Alfredo Ardisson Cirino Campos et al., 2025 (6)** | Y | Y | Y | Y | PY | PY | *Low* | Y | Y | Y | **High** | A number of concerns identified across domains, notably lack of formal RoB assessment |
| **Anggreni et al., 2025 (7)** | Y | Y | Y | PY | N | PY | *High* | Y | Y | Y | **High** | A number of concerns identified across domains, notably lack of supplementary searches |
| **Aucoin et al. 2020 (8)** | NI | PY | PY | Y | PY | N | *High* | PN | Y | Y | **High** | A number of concerns identified in domains 1-4 |
| **Aujla et al., 2024 (9)** | Y | Y | Y | Y | Y | Y | *Low* | Y | Y | Y | **Low** | Robust, transparent methodology with publication biased assessed |
| **Bagg et al., 2024 (10)** | PY | Y | PY | Y | PY | N | *High* | N | PY | PY | **High** | A number of concerns identified in domains 2-4 |
| **Balk et al. 2023a (11)** | Y | Y | PY | PN | Y | PY | *High* | PY | Y | Y | **Low** | The impact of concern was discussed |
| **Balk et al. 2023b (12)** | Y | Y | PY | PN | Y | PY | *High* | PY | Y | Y | **Low** | The impact of concern was discussed |
| **Baron et al. 2013 (13)** | PY | PY | PY | PY | PY | N | *High* | PN | Y | PN | **High** | A number of concerns identified in domains 1-4; only included studies that reported adverse events in the analysis |
| **Bell-Aldeghi et al. 2023 (14)** | PY | Y | PY | Y | PY | N | *High* | PN | Y | PY | **High** | A number of concerns identified in domains 1-4; only included studies that reported adverse events in the analysis |
| **Bilal et al. 2018 (15)** | Y | PY | PY | Y | PY | Y | *Low* | PN | Y | Y | **High** | A number of concerns or lack of information identified in domains 1-4 |
| **Briand et al. 2025 (16)** | Y | PY | PY | PY | NI | PY | *Unclear* | PY | Y | Y | **Unclear** | A number of concerns identified across domains, notably an absence of robustness analyses |
| **Brons et al., 2024 (17)** | Y | Y | Y | Y | N | PY | *High* | PY | Y | Y | **High** | A number of concerns identified across domains, notably no screening adjudication or data extraction QC |
| **Brozek et al. 2024 (18)** | Y | Y | PY | Y | PY | PY | *Low* | Y | Y | Y | **Low** | No concerns identified |
| **Buchlak et al. 2022 (19)** | PY | PY | PY | PY | PY | PY | *Low* | PN | PN | Y | **High** | A number of concerns or lack of information identified in domains 1-3 |
| **Buchlak et al. 2021 (20)** | PY | PY | PY | PN | PY | PN | *High* | PN | PY | Y | **High** | A number of concerns or lack of information identified in domains 1-4 |
| **Buchlak et al. 2020 (21)** | PY | PY | PY | PY | PY | PY | *Low* | PN | PY | Y | **High** | A number of concerns or lack of information identified in domains 1-3 |
| **Burger et al., 2025 (22)** | Y | Y | Y | Y | Y | Y | *Low* | Y | Y | Y | **Low** | Robust, transparent methodology using validated tools and GRADE assessment |
| **Canova et al., 2024 (23)** | Y | Y | Y | Y | N | PY | *High* | Y | Y | Y | **Low** | Robust, transparent methodology using validated tools for RoB assessment |
| **Carlson et al. 2023 (24)** | PY | PY | PY | Y | PY | N | *High* | PN | PY | Y | **High** | A number of concerns or lack of information identified in domains 3-4 |
| **Chamseddine et al. 2022 (25)** | PY | PY | PY | Y | PY | PY | *Low* | PN | Y | Y | **High** | A number of concerns or lack of information identified in domains 1-3 |
| **da Silva Mulder et al., 2024 (26)** | Y | Y | Y | Y | N | Y | *High* | Y | Y | Y | **Low** | Robust, transparent methodology |
| **da Silva et al., 2025 (27)** | Y | Y | Y | Y | N | PY | *High* | Y | Y | Y | **Low** | Robust, transparent methodology |
| **Das et al., 2024 (28)** | Y | Y | Y | Y | N | PY | *High* | Y | Y | Y | **Low** | Robust, transparent methodology |
| **de Gans et al. 2024 (29)** | PY | Y | PY | Y | PY | Y | *Low* | Y | Y | Y | **Low** | No concerns identified |
| **De Zwart & Ruis, 2024 (30)** | PY | PY | PY | Y | PY | N | *High* | PN | PY | Y | **High** | A number of concerns or lack of information identified in domains 1-4 |
| **Demirtas Yilmaz., 2025 (31)** | PY | Y | Y | Y | N | N | *High* | PN | Y | Y | **High** | Single-reviewer processes for study selection and data extraction, lack of supplementary search methods, and absence of formal risk-of-bias assessment |
| **Ducreux et al., 2025 (32)** | Y | Y | Y | Y | PY | Y | *Low* | Y | Y | Y | **Low** | Robust, transparent methodology |
| **Feng Q et al., 2025 (33)** | Y | Y | Y | Y | PY | PY | *Low* | Y | Y | Y | **High** | A number of concerns identified across domains, notably lack of formal RoB assessment |
| **Feng X et al., 2025 (34)** | Y | Y | Y | Y | PY | Y | *Low* | Y | Y | Y | **Low** | Robust, transparent methodology |
| **Franzoi et al. 2024 (35)** | Y | PY | PY | Y | PY | PY | *Low* | PN | Y | y | **High** | Concerns about restrictions applied in search strategies |
| **García-Torres et al., 2024 (36)** | Y | Y | Y | Y | PY | Y | *Low* | Y | Y | Y | **Low** | Robust, transparent methodology |
| **Ghozy et al., 2024 (37)** | Y | Y | Y | Y | Y | Y | *Low* | Y | Y | Y | **Low** | Robust, transparent methodology |
| **Glenn et al., 2025 (38)** | Y | Y | Y | Y | PY | Y | *Low* | Y | Y | Y | **Low** | Robust, transparent methodology |
| **Goldkuhle et al. 2018 (39)** | Y | PY | PY | Y | PY | PY | *Low* | Y | Y | Y | **Low** | No concerns identified |
| **Grinzinger et al., 2025 (40)** | Y | Y | Y | Y | N | PY | *High* | Y | Y | Y | **High** | A number of concerns identified across domains, notably lack of formal RoB assessment and single reviewer |
| **Halamoda‐Kenzaoui et al. 2022 (41)** | PY | PY | PY | NI | PY | N | *High* | PN | PY | Y | **High** | A number of concerns identified in domains 1-4 |
| **Hollands et al. 2019 (42)** | Y | Y | PY | Y | Y | Y | *Low* | Y | Y | Y | **Low** | No concerns identified |
| **Hosseini et al. 2023 (43)** | PY | PY | PY | Y | PY | N | *High* | PN | PY | Y | **High** | A number of concerns or lack of information identified in domains 1-4 |
| **Hsieh et al., 2024 (44)** | Y | Y | Y | Y | Y | Y | *Low* | Y | Y | Y | **Low** | Robust, transparent methodology |
| **Iaconisi et al., 2024 (45)** | Y | Y | Y | PY | N | N | *High* | PY | Y | Y | **High** | A number of concerns identified across domains, notably lack of formal RoB assessment |
| **Jackson et al. 2022 (46)** | Y | Y | PY | Y | Y | Y | *Low* | Y | Y | Y | **Low** | No concerns identified |
| **Jayasundara et al. 2024 (47)** | Y | PY | PY | Y | Y | PY | *Low* | PN | Y | Y | **High** | A number of concerns or lack of information identified in domains 1-3 |
| **Jayawardane et al., 2025 (48)** | Y | Y | Y | Y | N | PY | *High* | PY | Y | Y | **High** | A number of concerns identified across domains, notably lack of formal RoB assessment |
| **Kang et al., 2025 (49)** | Y | Y | Y | Y | Y | PY | *Low* | Y | Y | Y | **Low** | Robust, transparent methodology |
| **Karagiannis et al. 2019 (50)** | PY | PY | PY | PY | PY | PN | *High* | PN | Y | Y | **High** | A number of concerns identified in domains 2 and 4 |
| **Kendall et al., 2025 (51)** | Y | Y | Y | Y | Y | PY | *Low* | Y | Y | Y | **Low** | Robust, transparent methodology |
| **Kim & Cruz, 2022 (52)** | PY | PY | PY | PY | PY | N | *High* | PN | PY | Y | **High** | A number of concerns or lack of information identified in domains 1-4 |
| **Kumar et al., 2025 (53)** | Y | Y | Y | Y | PY | PY | *Low* | Y | Y | Y | **Low** | Robust, transparent methodology |
| **Lam et al. 2019 (54)** | PY | PY | PY | Y | PY | N | *High* | PN | PY | Y | **High** | A number of concerns or lack of information identified in domains 2-4 |
| **Landau et al., 2024 (55)** | Y | Y | Y | PY | N | N | *High* | PY | Y | Y | **Moderate** | A number of concerns identified across domains, notably lack of formal RoB assessment |
| **Liu Y et al., 2025 (56)** | Y | Y | Y | Y | Y | PY | *Low* | Y | Y | Y | **Low** | Robust, transparent methodology |
| **Lowe et al. 2021 (57)** | PY | Y | PY | Y | PY | PY | *Low* | Y | Y | Y | **Low** | The impact of identified concern was discussed by the authors |
| **Marin et al., 2024 (58)** | Y | Y | Y | Y | PY | PY | *Low* | Y | Y | Y | **Low** | Robust, transparent methodology |
| **McOwiti et al. 2024 (59)** | PY | PY | PY | Y | PY | N | *High* | PN | Y | Y | **High** | A number of concerns identified in domains 1-4 |
| **Meherali et al., 2025 (60)** | Y | PY | Y | PY | N | PY | *High* | PY | Y | Y | **High** | A number of concerns identified across domains, notably lack of analytical depth leading to limited |
| **Melinte et al., 2025 (61)** | Y | Y | Y | Y | Y | PY | *Low* | Y | Y | Y | **Low** | Robust, transparent methodology |
| **Merino-Barbancho et al., 2025 (62)** | PY | Y | PY | PY | N | PN | *High* | PY | Y | Y | **High** | A number of concerns identified across domains, notably lack of formal RoB assessment |
| **Miranda et al. 2021 (63)** | PY | PY | PY | Y | PY | N | *High* | PN | Y | Y | **High** | A number of concerns or lack of information identified in domains 2-4 |
| **Mushcab et al., 2025 (64)** | Y | Y | Y | Y | N | PY | *High* | PY | Y | Y | **Low** | Robust, transparent methodology with minor concerns due to heterogeneity |
| **Napolitano et al., 2022 (65)** | PY | PY | PY | Y | PY | N | *High* | PN | Y | Y | **High** | A number of concerns or lack of information identified in domains 1-4 |
| **Nogueira et al., 2025 (66)** | Y | Y | Y | Y | N | PY | *High* | PY | Y | Y | **Low** | Robust, transparent methodology with minor concerns due to heterogeneity |
| **Noteboom et al., 2024 (67)** | Y | Y | Y | PY | N | PY | *High* | PY | Y | Y | **Low** | Robust, transparent methodology with minor concerns due to heterogeneity |
| **Olaya-Mira et al., 2025 (68)** | Y | Y | Y | PY | N | PY | *High* | PY | Y | Y | **High** | A number of concerns identified across domains, notably lack of formal RoB assessment |
| **Petrolini-Mateus et al., 2025 (69)** | Y | Y | Y | PY | N | PY | *High* | PY | Y | Y | **Low** | Robust, transparent methodology with minor concerns due to heterogeneity |
| **Pillay et al. 2022 (70)** | PY | PY | PY | Y | PY | PY | *Low* | PN | Y | Y | **High** | Concern identified in domain 1 |
| **Pinna et al. 2020 (71)** | PY | PY | PY | Y | PY | PY | *Low* | PN | Y | Y | **High** | A number of concerns or lack of information identified in domains 1 and 3 |
| **Rakhshandehroo et al. 2023 (72)** | PY | PY | PY | Y | PY | PY | *Low* | PN | Y | Y | **Unclear** | No information about how many reviewers assessed the risk of bias |
| **Riaz et al. 2021 (73)** | PY | PY | PY | Y | Y | PY | *Low* | PN | Y | Y | **High** | A number of concerns or lack of information identified in domains 1-3 |
| **Robinson et al., 2024 (74)** | Y | PY | Y | PY | N | PY | *High* | PY | Y | Y | **High** | A number of concerns identified across domains, notably lack of formal RoB assessment |
| **Sarbout et al., 2025 (75)** | Y | Y | Y | PY | N | PY | *High* | Y | Y | Y | **High** | A number of concerns identified across domains, notably a single screener |
| **Shakeri Hossein Abad et al. 2021 (76)** | PY | PY | PY | Y | PY | N | *High* | PN | Y | Y | **High** | A number of concerns or lack of information identified in domains 1-4 |
| **Shemilt et al. 2013 (77)** | PY | PY | PY | PY | PY | N | *High* | PY | Y | Y | **Low** | The impact of identified concern was discussed by the authors |
| **Silva et al. 2022 (78)** | PY | PY | PY | Y | PY | N | *High* | PN | Y | Y | **High** | A number of concerns or lack of information identified in domains 1-4 |
| **Slebe et al. 2024 (79)** | PY | PY | PY | Y | Y | PN | *High* | PY | Y | Y | **Low** | The impact of identified concern was discussed by the authors |
| **Sorrentino et al., 2024a (80)** | Y | Y | Y | Y | N | PY | *High* | Y | Y | Y | **Low** | Robust, transparent methodology despite lack of sensitivity analyses |
| **Sorrentino et al., 2024b (81)** | Y | Y | Y | Y | N | PY | *High* | Y | Y | Y | **Low** | Robust, transparent methodology |
| **Spinelli et al. 2023 (82)** | PY | PY | PY | Y | PY | PN | *High* | PN | Y | Y | **High** | A number of concerns or lack of information identified in domains 1, 2, and 4 |
| **Steele et al., 2025 (83)** | Y | Y | Y | Y | PY | Y | *Low* | Y | Y | Y | **Low** | Robust, transparent methodology |
| **Sun et al., 2025 (84)** | Y | Y | Y | PY | N | PY | *High* | Y | Y | Y | **Low** | Robust, transparent methodology - minor concerns from lack of data extraction QC |
| **Susai et al., 2024 (85)** | Y | Y | Y | Y | N | N | *High* | PN | Y | Y | **High** | lack of supplementary search methods and no formal risk-of-bias assessment |
| **Talukdar et al., 2024 (86)** | Y | Y | Y | Y | Y | Y | *Low* | Y | Y | Y | **Low** | Robust, transparent methodology |
| **Teperikidis et al. 2023 (87)** | PY | PY | PY | Y | PY | PY | *Low* | PN | Y | Y | **High** | A number of concerns or lack of information identified in domains 1-3 |
| **Tun et al., 2025 (88)** | Y | PY | Y | Y | PN | PN | *High* | PN | Y | Y | **High** | Major concerns in the search strategy (limited databases) and lack of robustness analyses. |
| **Valizadeh et al., 2025a (89)** | Y | Y | Y | Y | PY | Y | *Low* | Y | Y | Y | **Low** | Robust, transparent methodology |
| **Valizadeh et al., 2025b (90)** | Y | Y | Y | Y | PY | Y | *Low* | Y | Y | Y | **Low** | Robust, transparent methodology |
| **Vallury et al. 2015 (91)** | PY | PY | PY | Y | PY | Y | *Low* | PN | Y | Y | **Unclear** | The impact of concern identified in domain 1 was discussed by the authors; not enough information for domain 3 |
| **van den Berg et al., 2025 (92)** | Y | Y | Y | Y | PY | Y | *Low* | Y | Y | Y | **Low** | Robust, transparent methodology - minor concerns from lack of risk-of-bias assessment |
| **Van Dijk et al. 2024 (93)** | PY | PY | PY | Y | PY | Y | *Low* | Y | Y | Y | **Low** | No concerns identified |
| **Visser et al., 2025 (94)** | Y | Y | Y | Y | PY | Y | *Low* | Y | Y | Y | **Low** | Robust, transparent methodology |
| **Vizcarra et al., 2024 (95)** | Y | PY | PY | PY | NI | Y | *Unclear* | Y | Y | Y | **Unclear** | Robust methodology, but absence of robustness analyses |
| **Voorn et al., 2025 (96)** | Y | Y | Y | Y | Y | Y | *Low* | Y | Y | Y | **Low** | Robust, transparent methodology |
| **Wen et al., 2025 (97)** | Y | Y | Y | Y | Y | Y | *Low* | Y | Y | Y | **Low** | Robust, transparent methodology |
| **Westendorp et al. 2023 (98)** | PY | PY | PY | Y | PY | Y | *Low* | PY | Y | Y | **Low** | The impact of identified concern was discussed by the authors |
| **Yamikan et al., 2025 (99)** | Y | Y | Y | Y | Y | Y | *Low* | Y | Y | Y | **Low** | Robust, transparent methodology |
| **Yappalparvi et al. 2025 (100)** | Y | PY | Y | PN | N | N | *High* | PN | Y | PY | **High** | Major methodological concerns throughout due to lack of risk of bias assessment and sesnsitivity analyses. |
| **Yazicioglu et al., 2025 (101)** | Y | Y | Y | Y | Y | Y | *Low* | Y | Y | Y | **Low** | Robust, transparent methodology |
| **Zamantakis et al., 2025 (102)** | Y | Y | Y | Y | N | PY | *High* | Y | Y | Y | **High** | A number of concerns identified across domains, notably lack of formal RoB assessment |
| **Zhu et al. 2023 (103)** | PY | PY | PY | Y | PY | Y | *Low* | PN | Y | Y | **High** | Concerns identified in domain 2 |
| **Crossingham et al 2021 (104)** | Y | Y | PY | Y | Y | Y | *Low* | Y | Y | Y | **Low** | No concerns identified |
| **Eun et al 2021 (105)** | PY | PY | PY | PY | Y | PY | *Low* | PN | Y | Y | **High** | A number of concerns or lack of information identified in domains 1-3 |
| **Foulquier et al 2018 (106)** | PY | PY | PY | Y | PY | N | *High* | PN | PY | Y | **High** | A number of concerns or lack of information identified in domains 1-4 |
| **Gaskins et al 2021 (107)** | PY | PY | PY | Y | PY | N | *High* | PN | PY | Y | **High** | A number of concerns identified in domains 2-4 |
| **Giummarra et al 2020 (108)** | PY | PY | PY | Y | PY | PY | *Low* | PY | Y | Y | **Low** | The impact of identified concern was discussed by the authors |
| **Rogers et al 2020 (109)** | PY | PY | PY | Y | Y | PN | *High* | PY | Y | Y | **Low** | The impact of identified concern was discussed by the authors |
| **Viner et al 2022 (110)** | PY | PY | PY | Y | PY | Y | *Low* | PN | Y | Y | **High** | Concerns identified in domain 1 |
| **Xiong et al 2018 (111)** | Y | PY | PY | PN | PY | PY | *High* | PN | PY | Y | **High** | A number of concerns or lack of information identified in domains 1-4 |
| **Yamamoto et al 2021 (112)** | Y | PY | PY | PN | Y | N | *High* | PN | PY | Y | **High** | A number of concerns identified in domains 1, 2, and 4 |

N, No; NI, No Information; PN, Probably No; PY Probably Yes; RoB, risk of bias; Y, Yes.

## Supplementary Table 4. Details of AI implementations and human involvement in included reviews.

| **Reference** | **AI tool** | **Sub-stage** | **Method of use** | **Human-in-the-loop** | |
| --- | --- | --- | --- | --- | --- |
| **Identified from database searches** | | | | |  |
| Aali et al. 2020 (1) | RobotReviewer | Risk of bias assessment | Automatically assesses the risk of bias of RCTs using the Cochrane Risk of Bias tool for Randomised Trials. | One reviewer checked and verified the assessment result. | |
| Agarwal et al. 2021 (2) | Cochrane RCT Classifier | Title/abstract screening | Predicts the probability that a record is an RCT. | Two reviewers independently screened titles and abstracts of records with a probability of being an RCT ≥10%, one reviewer screened those with a probability <10%. | |
| Al-Obeidat et al., 2024 (3) | Rayyan | Title/abstract screening | No details were provided. | Unclear | |
| Al-Sammarraie et al., 2025 (4) | GPT-4 Turbo | Data extraction | Extracts key features from research papers | Validated and corrected the AI-extracted data, refined prompts, resolved discrepancies, and reviewed final results | |
| Albuquerque et al., 2025 (5) | *NLP toolkit (developed in Python) | Search strategy development | Automatically searches scientific databases for relevant articles using keywords and inclusion criteria, and to remove duplicates based on DOI numbers. | Humans manually reviewed all studies identified by the AI search. | |
| Alfredo Ardisson Cirino Campos et al., 2025 (6) | Rayyan | Title/abstract screening | No details were provided. | Unclear | |
| Anggreni et al., 2025 (7) | NVivo | Data extraction | Assisted with data extraction by creating hierarchical codes | Data extracted and summarized manually | |
| Aucoin et al. 2020 (8) | Abstrackr | Title/abstract screening | Predicts the relevance of records and re-ranks abstracts for screening from most to least relevant. | Manually screened records with a probability of relevance ≥40%. | |
| Aujla et al., 2024 (9) | Nested-Knowledge Auto living semi-automated systematic review platform | Title/abstract screening | Provides a structured framework for undertaking literature reviews and meta-analyses and ensures rigorous,  transparent, and replicable procedures. | Resolved discrepancies and reviewed AI-assisted selections | |
| Bagg et al., 2024 (10) | Research Screener | Title/abstract screening | Predicts the relevance of records and re-ranks abstracts for screening from most to least relevant. | 1. A manually labeled training set; 2. One reviewer screened the prioritized records (stopping rule: no records were included from 2 consecutive subsets of 50 records). | |
| Balk *et al.* 2023a,b (11, 12) | Abstrackr | Title/abstract screening | No details were provided. | Two reviewers manually screened the abstracts with discrepancies resolved in team meetings or by a senior reviewer. | |
| Baron et al. 2013 (13) | *An automated text-mining tool | Title/abstract screening | Used to search for groups of words that are relevant to the review question. A weighted index was defined based on the frequency and place of occurrence of these words, which was used to select relevant articles. | The most relevant articles were manually screened. | |
| Bell-Aldeghi et al. 2023 (14) | *An automatic text classification algorithm (developed in Python) | Title/abstract screening  (In parallel with the manual process) | Used to automatically classify records as include and exclude based on the training set. | Performed after the manual process was completed. Used different proportions of the manually labeled abstracts as the training set to test performance. | |
| Bilal et al. 2018 (15) | RobotReviewer | Risk of bias assessment | No details were provided. | Unclear | |
| Briand et al. 2025 (16) | NVivo | Data extraction | Used for qualitative coding and clustering | Coding validated by coauthors | |
| Brons et al., 2024 (17) | ASReview | Title/abstract screening | Uses ML to arrange studies by relevance during title and abstract screening | Manual screening of all titles and abstracts | |
| Brozek et al. 2024 (18) | Laser AI | Data extraction | Automatically extracts data based on pre-defined vocabularies. | Human verification of extracted data. | |
| Buchlak et al. 2022 (19) | *NLP analysis using ML (developed in Python) | Title/abstract screening  (In parallel with the manual process) | The algorithm first identifies the keywords from included abstracts and a document classifier was trained to automate the screening process. | Manually labeled records as training set. | |
| Buchlak et al. 2021 (20) | *NLP analysis using ML (developed in Python) | Title/abstract screening  (In parallel with the manual process) | The algorithm first identifies the keywords from included abstracts and categorizes them into different topics; a document classifier was then trained to automate the screening process. | Manually labeled records as training set. | |
| Buchlak et al. 2020 (21) | *NLP analysis using ML (developed in Python) | Title/abstract screening  (In parallel with the manual process) | The algorithm first identifies the keywords from included abstracts and categorizes them into different topics. | Manual screening of records. | |
| Burger et al., 2025 (22) | ASReview | Title/abstract screening | Uses active learning to arrange articles by relevance | Manually labelled records as training set | |
| Canova et al., 2024 (23) | ASReview | Title/abstract screening | Uses active learning to arrange articles by relevance | Manually labelled records as training set | |
| Carlson et al. 2023 (24) | *DoCTER (ML and NLP) | Title/abstract screening | Groups records into different clusters using six different approaches. Clusters with seed references (manually labeled records) were selected. Records that were selected in three or more approaches were prioritized, and those that were only selected in one or two approaches went through additional ML approaches to prioritize relevant records. | 1. A manually labeled training set (483 studies); 2. Prioritized studies were manually screened. | |
|  | SWIFT-Active Screener | Title/abstract screening | Predicts the relevance of records and re-ranks records for screening from most to least relevant. | Prioritized studies were manually screened. | |
| Chamseddine et al. 2022 (25) | *A semi-automated NLP algorithm | Title/abstract screening | Used to identify abstracts that are relevant for penetrance, prevalence, both, or neither. | At least two reviewers independently screened the identified abstracts. | |
| da Silva Mulder et al., 2024 (26) | Rayyan | Title/abstract screening | Helped reviewers manage decisions and identify duplicates | Manual double-blind screening of identified articles | |
| da Silva et al., 2025 (27) | Rayyan | Title/abstract screening | Aided study selection | Manual double-blind screening of identified articles | |
| Das et al., 2024 (28) | Research Screener | Title/abstract screening | ML-based semi-automated title and abstract screening, ranking citations by relevance. | Two independent reviewers screened titles and abstracts within the AI platform, with results fed back to the AI for reranking. | |
| de Gans et al. 2024 (29) | ASReview | Title/abstract screening | 1. Based on prior decisions made by reviewers, predicts the relevance of records and re-ranks records for screening from most to least relevant. 2. An additional model was trained to search for any remaining relevant papers. | Two reviewers independently screened titles and abstracts (stopping rule: 150 irrelevant records) | |
| De Zwart & Ruis, 2024 (30) | ASReview | Title/abstract screening | Based on prior decisions made by reviewers, predicts the relevance of records and re-ranks records for screening from most to least relevant. | 1. A manually labeled training set (three articles);  2. Manual screening of records (stopping rule: 50 consecutive papers were excluded). | |
| Demirtas Yilmaz., 2025 (31) | Rayyan | Title/abstract screening | ML algorithms predicted inclusion/exclusion, detected duplicates, provided recommendations, and categorized studies | Manual reviewers set inclusion/exclusion criteria, filtered/tagged studies, reviewed and classified articles, ensured objectivity | |
| Ducreux et al., 2025 (32) | ASReview | Title/abstract screening | Used in parallel with manual reviewers to independently validate study selection, using a supervised method to rank and select relevant studies for inclusion. | Two manual reviewers independently performed literature search and study selection; AI results were compared with manual selection, and final inclusion required agreement across methods. Disagreements or uncertainties were resolved by discussion. | |
| Feng Q et al., 2025 (33) | Grobid - semi-automated workflow | Data extraction | Combined text mining with manual data extraction. | Manual data extraction. | |
| Feng X et al., 2025 (34) | BioMedGPT-LM-7B | Search strategy development | Model was used to generate a series of search strategies related to RCTs until two experts reached agreement. GPT then generate search strategies for conducting searches across multiple databases. | Experts reached agreement on search strategy. | |
|  |  | Risk of bias assessment | Model was trained with a set of RCTs already assessed by the Cochrane Risk of Bias Tool. The model was then applied to new RCTs to automatically assess risk of bias across domains. | AI results were compared with human assessment. | |
| Franzoi et al. 2024 (35) | ASReview | Title/abstract screening | Based on prior decisions made by reviewers, predicts the relevance of records and re-ranks records for screening from most to least relevant. | 1. A manually labeled training set (five articles);  2. Two reviewers independently screened titles and abstracts (stopping rule: 5% for the search database, 10–11% for the mega-meta databases); 3. A senior reviewer screened the excluded records. | |
| García-Torres et al., 2024 (36) | ChatGPT-4 and 4o | Data extraction | Data extraction was performed in parallel by two human reviewers and two versions of ChatGPT, each configured for review tasks. For the AI extraction, each prompt was run three times in independent conversations to address potential variability. | The results from all reviewers (human and AI) were compared, and discrepancies were resolved by a third human reviewer, who selected the most voted answer or made a final decision if needed. | |
| Ghozy et al., 2024 (37) | AutoLit (Nested Knowledge) | Title/abstract screening | No details provided | Screening and data extraction performed manually using the AutoLit (nested Knowledge) platform | |
|  |  | Data extraction |  |  |  |
| Glenn et al., 2025 (38) | ASReview | Title/abstract screening | Operates through active learning and a binary classification system, where reviewers iteratively label records as relevant or irrelevant, and the model updates predictions in real time. | All screening decisions made by human | |
| Goldkuhle et al. 2018 (39) | RobotReviewer | Data extraction | Automatically extracts data from RCTs (planned to use but no RCTs were identified) | Planned to compare data extraction and risk of bias assessment by RobotReviewer and a human reviewer, but no RCTs were identified. | |
|  |  | Risk of bias assessment | Automatically assesses the risk of bias of RCTs using the Cochrane Risk of Bias tool for Randomised Trials. |  |  |
| Grinzinger et al., 2025 (40) | scite.ai, consensus.ai. | Search strategy development | Used to supplement searches | Searches also conducted in traditional electronic databases | |
| Halamoda‐Kenzaoui et al. 2022 (41) | SWIFT-Review | Title/abstract screening | Automatic title/abstract screening. | 1. A manually labeled training set (ten records); 2. Manual screening of records of the lowest relevance. | |
|  | *segmenteR (developed in R) | Full-text screening | Used to extract the "material and methods" and "results" sections from full texts of articles. | No | |
|  | *A syntactic parsing tool | Full-text screening | Used to search for keywords that indicate relevance of the identified articles. A scoring system based on the keywords were designed and articles with a score equal to or above two were included. | No, except for the pre-defined keywords | |
|  |  | Data extraction | Used to extract keywords for outcomes of interest. | No, except for the pre-defined keywords | |
| Hollands et al. 2019 (42) | EPPI-Reviewer 4 | Title/abstract screening | 1. Based on prior decisions made by reviewers, predicts the relevance of records and re-ranks titles and abstracts for screening from most to least relevant. 2. Topic modeling was used to detect additional relevant records. Fifty topics and a series of ‘membership scores’ for each unscreened record were generated; unscreened record was allocated to the single topic that corresponded with its highest score. | 1. A manually labeled training set; 2. Two reviewers independently screened the prioritized records (stopping rule: 15 hours of duplicate screening without identifying any eligible records; equated to 1700 title-abstract records). 3. Reviewers manually ranked the topics based on their judgment of which topics could contain eligible studies. The ranking was combined with a data-generated one. Then, manual screening of a random sample of unscreened records was performed until the stopping rule was met. | |
| Hosseini et al. 2023 (43) | *A semi-automated NLP algorithm | Title/abstract screening | Used to identify abstracts that are relevant for penetrance, prevalence, both, or neither. | Two reviewers independently screened the identified abstracts. | |
| Hsieh et al., 2024 (44) | DistillerSR | Title/abstract screening | ML algorithm trained by human reviewers. | All citations were screened by at least one human reviewer, ML-flagged citations not selected for full text were rescreened by an independent reviewer, and two human reviewers assessed full-text eligibility | |
| Iaconisi et al., 2024 (45) | MySLR (Latent Dirichlet Allocation [LDA] algorithm) | Search strategy development | Automated topic-modeling system (LDA) was used to analyse the literature corpus | No details provided | |
|  |  | Screening | Filtered out irrelevant publications based on predefined criteria | Manual analysis of identified papers | |
|  |  | Data extraction | LDA was used to extract topics, keywords, and document-topic distributions from included papers. | Manual review of significant papers clustered around the identified topics | |
| Jackson et al. 2022 (46) | *An automated search strategy developed as part of the Human Behaviour Change Project (ML and NLP) | Literature search | No details were provided. | Used to complement the manual search strategies | |
| Jayasundara et al. 2024 (47) | Rayyan | Title/abstract screening | No details were provided. | A semi-automated procedure. | |
| Jayawardane et al., 2025 (48) | Rayyan | Title/abstract screening | ‘selection was conducted using the semi-automated tool Rayyan.’ No further details reported. | Manual, double-blind screening of all records. | |
| Kang et al., 2025 (49) | Rayyan | Title/abstract screening | Used to indicate articles with low relevance to this study. | Manual, double-blind screening of all records. | |
| Karagiannis et al. 2019 (50) | EPPI-Reviewer 4 | Title/abstract screening | Predicts the probability of a record being an RCT (trained by inputs from Cochrane Crowd) | One reviewer screened all titles and abstracts, a second reviewer screened abstracts with a probability of being an RCT ≥10%. | |
| Kendall et al., 2025 (51) | AutoLit (Nested Knowledge) | Data extraction | AutoLit (Nested Knowledge) platform used for data extraction. No further details reported. | Manual data extraction. | |
| Kim & Cruz, 2022 (52) | Leximancer | Data synthesis | Analyses full text and presents concept maps. | No | |
| Kumar et al., 2025 (53) | AutoLit (Nested Knowledge) | Search strategy development | To aid in deduplication of search results | Humans manually developed search queries, screened all records, performed tagging | |
|  |  | Title/abstract screening | Used for predicting which studies are relevant to this study, and providing suggestions for tagging. |  |  |
| Lam et al. 2019 (54) | SWIFT-Active Screener | Title/abstract screening | 1. Based on prior decisions made by reviewers, predicts the relevance of records and re-ranks records for screening from most to least relevant. 2. Predicts the number of eligible articles in the unscreened records. | Manual screening of prioritized records (stopping rule: estimated 95% recall) | |
|  | SWIFT-Review | Data extraction | Automatically extracts data on the outcome, baseline health, and comparison categories from the included abstracts, and tags articles with outcome categories. | Manual extraction of data that cannot be automatically extracted, including study length and sample size categories; manual verification of automatic tagging | |
| Landau et al., 2024 (55) | ChatGPT 3.5 | Data extraction | ChatGPT was used exclusively to generate variables and their definitions for the data extraction framework. | Human reviewers selected the final 13 variables, created the data collection form and validated AI-generated content. | |
| Liu Y et al., 2025 (56) | ASReview | Title/abstract screening | Predicting relevance after training on examples of relevant abstracts. | Human reviewers trained ASReview with relevant/irrelevant abstracts, then evaluated remaining abstracts. | |
| Lowe et al. 2021 (57) | Cochrane RCT Classifier | Title/abstract screening | Predicts the probability that a record is an RCT. | Two reviewers independently screened records with a probability of being an RCT ≥10%, one reviewer screened those with a probability <10%. | |
| Marin et al., 2024 (58) | Nested Knowledge, SciSpace, Elicit, Perplexity | Data extraction | Chain-of-Thought and Tree-of-Thought prompt-engineering techniques enhanced LLM reasoning for data extraction. | Two investigators independently verified all AI outputs | |
| McOwiti et al. 2024 (59) | ASReview | Title/abstract screening | No details were provided. | 1. A manually labeled training set (ten articles); 2. A semi-automated procedure. | |
| Meherali et al., 2025 (60) | EPPI reviewer | Data extraction | No details were provided. | Manual data extraction | |
| Melinte et al., 2025 (61) | Research Rabbitt | Supplementary searches (citation searching) | Used for backward snowballing to identify related articles. | Human reviewers conducted all database searching, screening, and eligibility assessment | |
| Merino-Barbancho et al., 2025 (62) | ASReview | Title/abstract screening | applied active learning to re-order articles by predicted relevance | Two independent researchers trained ASReview with relevant/irrelevant publications; results checked by a third reviewer | |
| Miranda et al. 2021 (63) | ASReview | Title/abstract screening | Based on prior decisions made by reviewers, predicts the relevance of records and re-ranks abstracts for screening from most to least relevant. | Manual screening of prioritized records. | |
| Mushcab et al., 2025 (64) | Elicit | Search strategy development | Search functionality used to identify relevant studies | Human reviewers conducted all database searching | |
| Napolitano et al., 2022 (65) | *A topic modeling tool (developed in R) | Title/abstract screening | Used to categorize abstracts into different topics. | Manually annotated keyword sets with a topic name. | |
|  | Semantic Scholar | Title/abstract screening | Used the AI function to identify Influential Citations received by each author, which was used as a ranking metric to prioritize records | Manual screening of the top 100 articles from each topic. | |
|  | *A ML framework | Title/abstract screening | Used to predict the probability that a preprint article will pass peer review, which was used as a metric to evaluate the relevance of the record. |  | |
| Nogueira et al., 2025 (66) | Rayyan | Title/abstract screening | No details reported | Two authors conducted all screening | |
| Noteboom et al., 2024 (67) | ASReview | Title/abstract screening | Used to train an active-learning model to re-order articles by predicted relevance | Reviewers labelled articles to train ASReview, screened ASReview-ranked records, resolved doubts by consensus, and involved additional reviewers for disagreements | |
| Olaya-Mira et al., 2025 (68) | Rayyan | Title/abstract screening | No details reported | Four reviewers screened all records in blind mode and resolved discrepancies through discussion. | |
| Petrolini-Mateus et al., 2025 (69) | ChatGPT 4 | Title/abstract screening | To support initial screening process for update search (no further details reported) | Manual double-blind screening of all studies | |
| Pillay et al. 2022 (70) | DistillerSR | Title/abstract screening | Based on prior decisions made by reviewers, predicts the relevance of records and re-ranks records for screening from most to least relevant. | Manual screening of prioritized records. | |
| Pinna et al. 2020 (71) | Rayyan | Title/abstract screening | No details were provided. | A semi-automated procedure. | |
| Rakhshandehroo et al. 2023 (72) | ASReview | Title/abstract screening | Predicts the relevance of records and re-ranks records for screening from most to least relevant (three rounds of screening with different active learning models). | 1. A manually labeled training set.  2. Two reviewers independently screened titles, abstracts, and full texts (stopping rule: 100 consecutive irrelevant records or only one relevant record in the last 25% of the 100 irrelevant records) | |
| Riaz et al. 2021 (73) | *LIvE platform | Study selection (sub-stage unclear) | Involves a ML-based RCT classifier. | Unclear (two pathways available: human-in-the-loop and AI-powered pathway) | |
|  |  | Data synthesis | Used to automate the process (details not provided). |  |  |
| Robinson et al., 2024 (74) | ChatGPT 4 (Bing chat) | Search strategy development | To support search strategy development, specifically to convert the MEDLINE strategy for Embase, Cochrane, and Google Scholar, and to refine the search | Two authors developed the initial strategy; all authors reviewed and amended the final strategy | |
| Sarbout et al., 2025 (75) | Elicit | Search strategy development | Used to find more eligible studies beyond those already obtained from PubMed. | Manual verification of all identified papers. | |
| Shakeri Hossein Abad et al. 2021 (76) | *NLP tools | Literature search | Language modeling and lexical association analysis were used to identify the context-sensitive terms and design the search strategy. | Used to complement the manually designed search strategy. | |
| Shemilt et al. 2013 (77) | EPPI-Reviewer 4 | Title/abstract screening | Predicts the relevance of records and re-ranks titles and abstracts for screening from most to least relevant. | 1. Manually screened a random sample to estimate the baseline inclusion rate; 2. One reviewer manually screened the prioritized records (465,80 records). | |
| Silva et al. 2022 (78) | ASReview | Title/abstract screening | Based on prior decisions made by reviewers, predicts the relevance of records and re-ranks titles and abstracts for screening from most to least relevant. | Manual screening of prioritized records. | |
| Slebe et al. 2024 (79) | ASReview | Title/abstract screening | Based on prior decisions made by reviewers, predicts the relevance of records and re-ranks titles and abstracts for screening from most to least relevant. | 1. One reviewer manually screened the prioritized records (stopping rule: exclusion of 300 consecutive records); 2. Records included by the first reviewer and a random sample of ~200 records that were excluded were checked by another two reviewers. | |
| Sorrentino et al., 2024a (80) | Rayyan | Title/abstract screening | AI features used for screening – no further details reported | Five reviewers screened all titles and abstracts in Rayyan | |
| Sorrentino et al., 2024b (81) | Rayyan | Title/abstract screening | AI features used for screening – no further details reported | Three reviewers screened all titles and abstracts in Rayyan | |
| Spinelli et al. 2023 (82) | Rayyan | Title/abstract screening | Based on prior decisions made by reviewers, predicts the relevance of unscreened records. | A semi-automated procedure. | |
| Steele et al., 2025 (83) | Abstrackr | Title/abstract screening | ML algorithm used to assist abstract screening and determine when to stop double screening based on predicted relevance scores. | Two reviewers conducted double screening; humans stopped double screening when ML predicted relevance score of the remaining unscreened papers was below 0.40 | |
| Sun et al., 2025 (84) | ASReview | Title/abstract screening | Used ML to prioritize records based on predicted relevance | One reviewer screened all titles/abstracts; a second reviewer manually screened 15% (increasing to 30% if agreement <80%). Disagreements were resolved by consensus. | |
| Susai et al., 2024 (85) | CADIMA | Title/abstract screening | Automated screening of duplicate articles to eliminate technical bias | Three independent researchers reviewed and screened articles; institutional review board screened for observer bias. | |
| Talukdar et al., 2024 (86) | Litmaps | Supplementary searches (snowballling) | AI-assisted citation discovery was used to identify additional relevant studies via its “Discover” feature after traditional database searching. | Humans screened all Litmaps-identified articles and determined final inclusion. | |
| Teperikidis et al. 2023 (87) | ChatGPT | Literature search | Prompted to generate the search strategy. | ChatGPT was prompted by reviewers. | |
|  |  | Title/abstract screening  (in parallel with the manual process) | Prompted to perform screening. |  |  |
|  |  | Data extraction | Prompted to generate a data extraction table based on PICO criteria. |  |  |
|  |  | Risk of bias assessment  (in parallel with the manual process) | Prompted to perform quality assessment using AMSTAR 2.0. |  |  |
|  |  | Data synthesis | Prompted to generate individual study summaries and write the article. |  |  |
| Tun et al., 2025 (88) | Elicit | Data extraction | Used to screen and analyse abstracts. No further details reported. | Manual screening of identified studies | |
| Valizadeh et al., 2025a (89) | AutoLit (Nested Knowledge) | Title/abstract screening | Facilitated deduplication, screening, and data extraction. No further details reported. | No further details reported. | |
|  |  | Data extraction |  |  |  |
| Valizadeh et al., 2025b (90) | AutoLit (Nested Knowledge) | Title/abstract screening | Facilitated deduplication, screening, and data extraction. No further details reported. | No further details reported. | |
|  |  | Data extraction |  |  |  |
| Vallury et al. 2015 (91) | Nvivo | Full-text screening | Automatically searches full texts for keywords and their synonyms | Manual screening of full texts identified through the text-mining procedure. | |
| van den Berg et al., 2025 (92) | ASReview | Title/abstract screening | Active learning algorithm was used for title/abstract screening | The model was trained on 7 manually chosen relevant studies and 7 irrelevant ones, which ASReview suggested, and humans confirmed as irrelevant. Stopping rule: 100 subsequent irrelevant records. | |
| Van Dijk et al. 2024 (93) | ASReview | Title/abstract screening | Predicts the relevance of records and re-ranks titles and abstracts for screening from most to least relevant. | 1. A manually labeled training set (three relevant and three irrelevant articles); 2. Manual screening of prioritized records (stopping rule: 100 subsequent irrelevant records). | |
| Visser et al., 2025 (94) | ASReview | Title/abstract screening | ML-driven ranking of studies by relevance based on iterative human-labelled input | Two reviewers screened titles/abstracts independently using ASReview; disagreements were resolved by a third reviewer. Stopping rule: 100 subsequent irrelevant records. | |
| Vizcarra et al., 2024 (95) | Python and ChatGPT-4 | Title/abstract screening | Python code, finetuned with ChatGPT-4, used for semi-automated title/abstract screening, duplicate removal, and inclusion suggestion | Two authors manually reviewed abstracts, with 20% of exclusions checked by a reviewer. | |
| Voorn et al., 2025 (96) | ASReview | Title/abstract screening | Active learning algorithm was used for title/abstract screening, continuously re-ranking records by likely relevance. | Two reviewers independently screened all titles/abstracts in ASReview, manually labelled training examples. Stopping rule: 100 subsequent irrelevant records. | |
| Wen et al., 2025 (97) | ASReview | Title/abstract screening | Active learning algorithm was used for title/abstract screening, continuously re-ranking records by likely relevance. | Two authors independently screened articles marked as relevant by the AI tool | |
| Westendorp et al. 2023 (98) | ASReview | Title/abstract screening | Predicts the relevance of records and re-ranks records for screening from most to least relevant. | 1. A manually labeled training set: 20% of all records done by two independent reviewers); 2. One reviewer screened the prioritized records (stopping rule: 150 irrelevant records). | |
| Yamikan et al., 2025 (99) | Rayyan | Title/abstract screening | Rayyan AI-Powered Tool used for screening. No further details reported. | Two reviewers manually screened titles and abstracts blind. | |
| Yappalparvi et al. 2025 (100) | AutoLit (Nested Knowledge) | Title/abstract screening | Nested Knowledge platform was used for screening and data extraction. No further details reported. | Manual screening and review of all articles. | |
|  |  | Data extraction |  |  |  |
| Yazicioglu et al., 2025 (101) | Rayyan | Title/abstract screening | No details provided | All reviewers independently screened titles and abstracts in Rayyan; disagreements were resolved by consensus | |
| Zamantakis et al., 2025 (102) | Semi-automated text mining and natural language processing (custom) | Title/abstract screening | Text mining and natural language processing used to screen articles for eligibility | After automated exclusion, a team of researchers manually screened titles and abstracts and resolved discrepancies | |
| Zhu et al. 2023 (103) | ASReview | Title/abstract screening | Predicts the relevance of records and re-ranks records for screening from most to least relevant. | 1. A manually labeled training set (ten articles done by one reviewer); 2. The first reviewer manually screened all titles and abstracts; 3. A second reviewer screened titles and abstracts excluded by the first reviewer (stopping rule: fifty successively excluded records). | |
| **Identified from hand searches** | | | | |  |
| Crossingham et al 2021 (104) | Cochrane RCT Classifier | Title/abstract screening | Predicts the probability that a record is an RCT. | Four reviewers screened the titles and abstracts of records predicted to be RCTs. | |
| Eun et al 2021 (105) | *A ML approach (developed in Python) | Title/abstract screening  (in parallel with the manual process) | Two maximum entropy classifiers and a metric K-means clustering model were trained to predict and select relevant articles. | A manually labeled training set (140 articles). | |
| Foulquier et al 2018 (106) | *BIBOT (NLP; developed in Python) | Title/abstract screening | Used to filter records based on different criteria (such as year of publication and language) and text analysis to identify records with terms from the validation lists. | No | |
| Gaskins et al 2021 (107) | Rayyan | Title/abstract screening  (post-protocol) | Manually labeled records were uploaded to Rayyan, which predicts the relevance of records excluded in the manual process. | One reviewer manually screened the top 200 records by relevance. | |
| Giummarra et al 2020 (108) | Abstrackr | Title/abstract screening | Based on prior decisions made by reviewers, predicts the relevance of records and re-ranks records for screening from most to least relevant. | 1. Reviewer 1 manually screened all records; 2. Reviewer 2 screened records labeled as relevant until no further records were predicted to be relevant. | |
|  | Wordstat | Full-text screening | Automatically searches full texts for keywords and their synonyms. | 1. Keywords were defined and developed through consultations with experts; 2. Reviewer 1 manually screened all full text papers; 3. Reviewer 2 screened all full texts identified through the text-mining procedure. | |
|  | QDA Miner |  |  |  | |
| Rogers et al 2020 (109) | Rayyan | Title/abstract screening | No details were provided. | A semi-automated process. | |
| Viner et al 2022 (110) | EPPI-Reviewer 4 | Title/abstract screening | Predicts the relevance of records and re-ranks records for screening from most to least relevant and establishes a threshold below which records are unlikely to be relevant. | 1. A manually labeled training set (1500 articles); 2. Two researchers independently screened identified records. | |
| Xiong et al 2018 (111) | *A ML approach (developed in R) | Title/abstract screening  (in parallel with the manual process) | Records were sorted into different clusters. A ML model was trained to identify the most relevant clusters. | 1. A manually labeled training set (139 articles); 2. Manual screening of the identified clusters. | |
| Yamamoto et al 2021 (112) | RobotAnalyst | Title/abstract screening | Predicts the relevance of records and re-ranks records for screening from most to least relevant. | 1. A manually labeled training set; 2. Two reviewer screened the prioritized records. | |

RCT, randomized controlled trial.

## Supplementary Table 5 HTA agency and methodological body recommendations on use of AI in SLRs.

|  | **Document** | **Search strategy** | **Study selection** | **Data extraction** | **Risk of bias assessment** |
| --- | --- | --- | --- | --- | --- |
| **HTA agencies** | | | | | |
| **Canada’s Drug Agency 2025 (113)** | Position Statement on the Use of Artificial Intelligence in the Generation and Reporting of Evidence | ✔ ML methods and LLM prompts to generate search strategies | ✔ ML methods and LLM prompts to automate the classification of studies and screening of primary and full-text records to identify eligible studies | ✔ LLM to automate data extraction and synthesize extracted data |  |
| **Institut für Qualität und Wirtschaftlichkeit im Gesundheitswesen 2023 (114)** | General Methods | ✔ Text mining to develop search terms | ✔ Validated ML-based study classifiers  ? Other ML tools (e.g., for prioritization) can be tried |  |  |
| **National Institute for Health and Care Excellence 2024 (115)** | Position Statement on the Use of Artificial Intelligence in the Generation | ✔ Frequency analysis and text mining to develop search terms (e.g., PubReMiner, Medline Ranker) | ✔ Priority screening ML tools (cannot recommend the stopping rule)  ✔ Validated ML-based study classifiers (e.g., Cochrane RCT classifier) |  |  |
| **EUnetHTA 2019* (116)** | Methodological guidelines: Process of information retrieval for systematic reviews and health technology assessments on clinical effectiveness | ✔ Frequency analysis and text mining to develop search terms | ✔ Priority screening ML tools (cannot recommend the stopping rule)  ✔ Validated ML-based study classifiers (e.g., Cochrane RCT classifier) |  |  |
| **Methodological bodies** | | | | | |
| **Higgins *et al.* 2024 (117)** | Cochrane Handbook for systematic reviews of interventions |  | ✔ Validated ML-based study classifiers (e.g., Cochrane RCT classifier, EPPI-Reviewer, RobotReviewer, DistillerSR)  ✔ Priority screening ML tools (stopping rule not validated, thus only recommends semi-automation)  ✔ NLP tools to highlight sentences and keywords when screening  ? LLMs (e.g., ChatGPT) may become useful in the future | ? Inadequate evidence, but can be used to check the manually extracted data | ? Semi-automation using ML tools, but reliability uncertain |
| **JBI (118)** | JBI Manual for Evidence Synthesis | ✔ Generative AI tools (e.g. ChatGPT) for finding these seed references  ✔ ChatGPT and other tools like perplexity.ai or consensus.app for suggesting keyword synonyms |  |  |  |

* EUnetHTA is a collaborative network established by HTA agencies across Europe

LLM, large language model; ML, machine learning; NLP, natural language processing; RCT, randomized controlled trial.

## Supplementary Table 6. Reported advantages and disadvantages of AI tools for SLRs.

| **Reference** | **AI tool** | **Stage** | **Advantage** | **Disadvantage** | **Notes** |
| --- | --- | --- | --- | --- | --- |
| **Identified from database searches** | | | | | |
| Aali *et al.* 2020 (1) | RobotReviewer | Risk of bias assessment | Saves time and resources, maintains review quality | N/A |  |
| Albuquerque *et al*. 2025 (5) | *NLP toolkit (developed in Python) | Search strategy development | Improved efficiency in literature searching, reduced manual workload, and enabled rapid identification of relevant studies for systematic review. | N/A |  |
| Aucoin *et al.* 2020 (8) | Abstrackr | Title/abstract screening | Rapid screening | N/A | Inherent inaccuracy of ML of Abstrackr (~4.2% of studies are incorrectly identified for exclusion), so manual screening still needed to reduce the risk of missing relevant studies (Gates *et al.* 2018) |
| Bagg *et al.* 2024 (10) | Research Screener | Title/abstract screening | N/A | Risk of missing relevant articles |  |
| Baron *et al.* 2013 (13) | *An automated text-mining tool | Title/abstract screening | Good precision | Manual full-text screening still required |  |
| Bell-Aldeghi *et al.* 2023 (14) | *An automatic text classification algorithm (developed in Python) | Title/abstract screening | Good performance (99% specificity, 86.7% sensitivity) | N/A |  |
| Buchlak *et al.* 2022 (19) | *NLP analysis using ML (developed in Python) | Title/abstract screening | Moderate performance; potentially reduces screening time for future reviews on similar topics | N/A |  |
| Buchlak *et al.* 2021 (20) | *NLP analysis using ML (developed in Python) | Title/abstract screening | Some models had good performance | Others had unsatisfactory performance |  |
| de Gans *et al.* 2024 (29) | ASReview | Title/abstract screening | Aid decision-making, good performance | The additional model using Sentence BERT with logistic regression is more computationally intensive |  |
| de Zwart & Ruis, 2024 (30) | ASReview | Title/abstract screening | Faster screening, reliable | Error rate was unknown | The tool does not provide an accurate estimation of the system’s error rate (van de Schoot *et al.* 2021) |
| Ducreux *et al.* 2025 (32) | ASReview | Title/abstract screening | The AI-powered method was described as an efficient, quicker, and easier method of selection than the “manual” method. | N/A |  |
| Feng Q *et al.* 2025 (33) | Grobid - semi-automated workflow | Data extraction | The hybrid approach (text mining plus manual extraction) was described as sensitive, precise, and efficient, and has been widely used in systematic reviews. | N/A | Despite optimizing the algorithm and manually curating the extracted data, there were inaccuracies in data extraction; however, the  false positivity and false negativity of data extraction were all at or below 8%. |
| Franzoi *et al.* 2024 (35) | ASReview | Title/abstract screening | N/A | Risk of missing relevant articles and rank-order bias (the record ranking can affect decision-making, but was mitigated by multiple rounds of verifications) | There is a possibility that some relevant articles were missed; rank-order bias (when the arrangement of papers can influence decision-making; mitigated with a three-phase screening process) (Gargon *et al.* 2019; Norman 2020) |
| García-Torres et al. 2024 (36) | ChatGPT-4 and 4o | Data extraction | AI achieved a high level of accuracy in identifying key categories. | GPT-4 displayed a slightly higher error rate (8.6%) compared to human reviewers and GPT-4o, particularly in complex categories | GPT-4 achieved 91.4% correct responses (i.e., most voted responses) with an error rate of 8.6%. GPT-4o demonstrated higher accuracy, with 97.1% correct responses and a 2.9% error rate. Human reviewers achieved 95.7% and 97.1% correct responses, with error rates of 4.3% and 2.9%. |
| Glenn et al. 2025 (38) | ASReview | Title/abstract screening | Improved efficiency with substantial reductions in screening workload | N/A |  |
| Goldkuhle *et al.* 2018 (39) | RobotReviewer | Data extraction | N/A | Two non-RCTs incorrectly labeled as RCTs |  |
| Halamoda‐Kenzaoui *et al.* 2022 (41) | SWIFT-Review | Title/abstract screening | Faster screening, automatic exclusion of irrelevant records | N/A |  |
|  | *segmenteR (developed in R) | Full-text screening | The first tool to extract a specific section from articles | Did not work in 85 documents (out of 750) |  |
|  | *A syntactic parsing tool | Full-text screening | N/A | Difficulty in pre-defining keywords; did not differentiate positive and negative statements; could not perform detailed analysis |  |
|  |  | Data extraction | N/A | N/A |  |
| Hollands *et al.* 2019 (42) | EPPI-Reviewer 4 | Title/abstract screening | Topic modeling plus active learning can increase recall; reduce manual workload | N/A |  |
| Hosseini *et al.* 2023 (43) | *A semi-automated NLP algorithm | Title/abstract screening | N/A | Risk of missing relevant articles |  |
| Iaconisi et al. 2024 (45) | MySLR (Latent Dirichlet Allocation [LDA] algorithm) | Search strategy development | Reduces manual effort and improves consistency and accuracy by identifying key themes and concepts within the body of research | Misclassification of 36 papers, showing reduced precision |  |
|  |  | Screening |  |  |  |
|  |  | Data extraction |  |  |  |
| Jackson *et al.* 2022 (46) | *An automated search strategy developed as part of the Human Behaviour Change Project (ML and NLP) | Literature search | Identified additional studies | N/A |  |
| Karagiannis *et al.* 2019 (50) | EPPI-Reviewer 4 | Title/abstract screening | Accurate exclusion of non-RCTs, reduce workload, faster screening when a large number of records need to be screened | N/A |  |
| Lam *et al.* 2019 (54) | SWIFT-Active Screener | Title/abstract screening | Saves time and resources | A potential trade-off between time commitment and recall |  |
|  | SWIFT-Review | Data extraction | Save time and resources | Could not automatically extract data on study length and sample size |  |
| Miranda *et al.* 2021 (63) | ASReview | Title/abstract screening | Useful for prioritization | N/A |  |
| Napolitano *et al.* 2022 (65) | Semantic Scholar | Title/abstract screening | Facilitate screening when a large number of records need to be screened by selecting the most impactful studies | N/A |  |
|  | *A ML framework | Title/abstract screening |  |  |  |
| Rakhshandehroo *et al.* 2023 (72) | ASReview | Title/abstract screening | Reduce workload (identified more than 20,000 records) | Risk of missing relevant articles |  |
| Riaz *et al.* 2021 (73) | *LIvE platform | Study selection (sub-stage unclear) | Faster evidence synthesis and interactive result presentation | N/A |  |
|  |  | Data synthesis |  |  |  |
| Shakeri Hossein Abad *et al.* 2021 (76) | *NLP tools | Literature search | Identified terminology in a more consistent way | N/A |  |
| Shemilt *et al.* 2013 (77) | EPPI-Reviewer 4 | Title/abstract screening | Facilitate screening when a large number of records need to be screened | N/A |  |
| Silva *et al.* 2022 (78) | ASReview | Title/abstract screening | Save time, reduce workload | N/A |  |
| Spinelli *et al.* 2023 (82) | Rayyan | Title/abstract screening | Faster screening | N/A |  |
| Teperikidis *et al.* 2023 (87) | ChatGPT | Literature search | Generated an appropriate search string | N/A |  |
|  |  | Title/abstract screening | N/A | Did not produce reliable screening results when provided the review question and PICO criteria (had to prompt it to extract the PICO elements and then ask it to make judgment, which produced reliable results) |  |
|  |  | Data extraction | Successfully generated a data extraction table | N/A |  |
|  |  | Risk of bias assessment | N/A | Unreliable and produced higher scores than human reviewers |  |
|  |  | Data synthesis | Successfully generated study summaries and wrote the article | N/A |  |
| Westendorp *et al.* 2023 (98) | ASReview | Title/abstract screening | Saves time, prevents human error and bias | N/A |  |
| Zhu *et al.* 2023 (103) | ASReview | Title/abstract screening | N/A | Small risk of missing relevant articles | There is currently no evidence-based terminal point for article screening by the second reviewer using ASReview, potentially (although unlikely) excluding relevant records |
| **Identified from hand searches** | | | | | |
| Eun *et al.* 2021 (105) | *A ML approach (developed in Python) | Title/abstract screening | Reduce workload, good performance | N/A |  |
| Foulquier *et al.* 2018 (106) | *BIBOT (NLP; developed in Python) | Title/abstract screening | Enabled a broad search of a large number of records | N/A |  |
| Gaskins *et al.* 2021 (107) | Rayyan | Title/abstract screening | Increase recall, efficient, accurate, user-friendly, free | N/A |  |
| Giummarra *et al.* 2020 (108) | Abstrackr | Title/abstract screening | Faster screening, reduce workload | Small risk of missing relevant articles |  |
|  | Wordstat | Full-text screening | Reduce workload (from 555 to 367 after text mining) |  |  |
|  | QDA Miner |  |  |  |  |
| Rogers *et al.* 2020 (109) | Rayyan | Title/abstract screening | Faster screening | N/A |  |
| Viner *et al.* 2022 (110) | EPPI-Reviewer 4 | Title/abstract screening | Enabled a broad search of a large number of records | Risk of missing relevant articles |  |
| Xiong *et al.* 2018 (111) | *A ML approach (developed in R) | Title/abstract screening | Good performance, reduce workload (87% reduction), more efficient and objective | N/A |  |

ML, machine learning; N/A, not available; NLP, natural language programming; PICO, Population, Intervention, Comparator, Outcomes; RCT, randomized controlled trial.
